# Functional Characterization of Cotton C-Repeat Binding Factor Genes Reveal Their Potential Role in Cold Stress Tolerance

**DOI:** 10.3389/fpls.2021.766130

**Published:** 2021-12-08

**Authors:** Jiangna Liu, Richard Odongo Magwanga, Yanchao Xu, Tingting Wei, Joy Nyangasi Kirungu, Jie Zheng, Yuqing Hou, Yuhong Wang, Stephen Gaya Agong, Erick Okuto, Kunbo Wang, Zhongli Zhou, Xiaoyan Cai, Fang Liu

**Affiliations:** ^1^Chinese Academy of Agricultural Sciences (ICR, CAAS)/State Key Laboratory of Cotton Biology, Institute of Cotton Research, Anyang, China; ^2^School of Agricultural Sciences, Zhengzhou University (SBPMAS), Zhengzhou, China; ^3^School of Biological, Physical, Mathematics and Actuarial Sciences, Jaramogi Oginga Odinga University of Science and Technology (JOOUST), Bondo, Kenya

**Keywords:** CBF4, transcription factors, cold tolerance, overexpression, cotton

## Abstract

Low temperature is a common biological abiotic stress in major cotton-growing areas. Cold stress significantly affects the growth, yield, and yield quality of cotton. Therefore, it is important to develop more robust and cold stress-resilient cotton germplasms. In response to climate change and erratic weather conditions, plants have evolved various survival mechanisms, one of which involves the induction of various stress responsive transcript factors, of which the C-repeat-binding factors (CBFs) have a positive effect in enhancing plants response to cold stress. In this study, genomewide identification and functional characterization of the cotton CBFs were carried out. A total of 29, 28, 25, 21, 30, 26, and 15 proteins encoded by the *CBF* genes were identified in seven *Gossypium* species. A phylogenetic evaluation revealed seven clades, with Clades 1 and 6 being the largest. Moreover, the majority of the proteins encoded by the genes were predicted to be located within the nucleus, while some were distributed in other parts of the cell. Based on the transcriptome and RT-qPCR analysis, *Gthu17439* (*GthCBF4*) was highly upregulated and was further validated through forward genetics. The *Gthu17439* (*GthCBF4*) overexpressed plants exhibited significantly higher tolerance to cold stress, as evidenced by the higher germination rate, increased root growth, and high-induction levels of stress-responsive genes. Furthermore, the overexpressed plants under cold stress had significantly reduced oxidative damage due to a reduction in hydrogen peroxide (H_2_O_2_) production. Moreover, the overexpressed plants under cold stress had minimal cell damage compared to the wild types, as evidenced by the Trypan and 3,3′-Diaminobenzidine (DAB) staining effect. The results showed that the *Gthu17439* (*GthCBF4*) could be playing a significant role in enhancing cold stress tolerance in cotton and can be further exploited in developing cotton germplasm with improved cold-stress tolerance.

## Introduction

Cotton is a thermophilic crop and is more sensitive to low temperature (Hemantaranjan et al., 2014); China being the major cotton-growing country globally, the site specific regions within China, such as Xinjiang, is often affected by cold, which lasts for more than a half growing of cotton plants, which results in negative effects on plant growth and development (Rihan et al., 2017). Cold stress leads to inhibition of seed germination, reduction of plant growth, and reproduction, as well as a decrease in crop yield and quality (Körner, 2016). However, many crops, such as rice (*Oryza sativa*), maize (*Zea mays*), tomato (*Solanum lycopersicum*), soybean (*Glycine max*), and cotton (*Gossypium hirsutum*), do lack the ability to adapt to environments with low temperature, thus highly adaptive to tropical or subtropical regions (Xiong et al., 2002; Wang et al., 2017; Kumar Verma et al., 2018). In order to deter the emergence of the adverse effects, plants have evolved complex mechanisms to resist cold stress by integrating the plants’ transcription factors (Diouf et al., 2017; Lu et al., 2018; Magwanga et al., 2018b,^2019^). Through the integration of the physiological, biochemical, and molecular regulatory mechanisms into stress detection, signal transduction, gene expression, and metabolic modifications, plants have been found to have survival strategy for reducing or preventing the effects of cellular oxidative damage (Thomashow, 1999).

In order to understand the effect of cold stress on plants, it is important to distinguish between cold (0–15°C) and freezing/chilling stress (<0°C). Cold stress leads to metabolic injury, destruction of the stability of protein complexes, inhibition of the metabolic pathways and various cellular processes in varying degrees, and eventually, damage to the photosynthetic processes (Bruce et al., 2007; Colebrook et al., 2014; Zhao et al., 2018). Freezing/chilling temperature promotes the ice formation in intercellular spaces of plant tissues, significantly reduces the efficiency of photosynthesis, and increases the release of reactive oxygen species (ROS). Increased release of ROS leads to chloroplast membrane oxidation; moreover, increased levels of ROS initiate stress-signal mechanism in plants, which significantly alter gene expression at chloroplast and nucleus levels, thereby promoting lipid homeostasis, thereby increasing plants adaptation to chilling stress (Liu X. et al., 2018). Furthermore, when ice crystals are deposited on the cell wall, the extracellular water potential decreases, and the cell membrane is destroyed, resulting in serious cell dehydration (Uemura, 2014). The most common phenomenon to resist the adverse effects of cold is acclimatization through the evolution of stress-responsive genes, for instance, the C-repeat-binding factors (CBFs), being the CBF1 and CBF3 play a significant role in cold acclimation-dependent freezing tolerance (Zhao et al., 2016). Many plants have increased freezing resistance after a period of low non-freezing temperature, a phenomenon known as cold acclimation (Kumar et al., 2010; Hussain et al., 2018). Cold acclimation is an effective way to increase plant-freezing resistance after a period of low non-freezing temperature (Shi et al., 2018). When plants are exposed to chilling conditions, the plants do mobilize the cold-response genes (COR), which then activate.

C-repeat binding factors (CBFs), followed by the accumulation of cryoprotectants, results in the acquisition of freezing tolerance (Thomashow, 1999). When plants are exposed to non-freezing cold condition, the CBFs are rapidly induced by low temperature, and then the downstream target gene *COR* becomes activated (Zhang and Huang, 2010). In addition, as the expression of CBF is regulated by light quality, biological clock, and photoperiod, it is necessary to understand the daily and seasonal regulations of the *CBF* genes (Gong et al., 2020). The CBF/dehydration-responsive element binding factor 1 (CBF/DREB1) is well-studied as an integral transcription factor in the cold regulatory pathway. It is an adaptive response where plants increase freezing tolerance after exposure to low non-freezing temperatures (Liu et al., 1998). The CBF/DREB protein, a DNA-binding protein belonging to the AP2/ERF superfamily, was first identified in *Arabidopsis thaliana* as a cold (LT)-induced transcription factor (Jin Y. et al., 2018). The APETALA2/ethylene-responsive element binding factor (AP2/ERF) family is a large class of plant-specific transcription factors, including AP2, RAV, ERF, and CBF (Haake, 2002; Jin J. H. et al., 2018). The expression of *CBF* gene was very low at room temperature but increased rapidly within 15 min of plants exposure to cold stimulation (Wang et al., 2017; Shi et al., 2018). During cold acclimation, mobilization of the CBF activated cold response (*COR*) gene and increased accumulation levels of the cryoprotectants do significantly enhance the cold tolerance in plants (Thomashow, 1999). The *CBF/DREB* gene is activated by a *CBF* expression (ICE) inducer through specific binding of *cis*-elements to MYC (c-Myc, L-Myc, S-Myc, and N-Myc) in the promoter region (Chinnusamy et al., 2003; Jin Y. et al., 2018; Shi et al., 2018).

The cold reaction pathway of ICE-CBF-COR has been proved to be the effective defense mechanism to cold stress (Wang et al., 2017). Moreover, the known six members of the CBF family members CBF1/DREB1C, CBF2/DREB1B, and CBF3/DREB1A are induced by cold stress, while CBF4/DREB1D, DREB1E/DDF2, and DREB1F/DDF1 are induced by osmotic stresses (Ma et al., 2014). Furthermore, the CBF1, CBF2, and CBF3 are arranged in tandem in an 8.7-kb region of the short arm of chromosome 4 in Arabidopsis (Jin Y. et al., 2018). All the three CBF Transcription factors perhaps act as the activators of the expression of downstream *CORs* genes, but with different functions (Siddiqua and Nassuth, 2011). The CBF1/2/3 triple mutant Arabidopsis seedlings obtained by CRISPR/Cas9 technology are more sensitive to low temperature than the single mutant CBF2 and CBF3, as well as the double mutant CBF1/3 (Zhao et al., 2016), which is an indication that *CBF* genes play a significant role in enhancing cold stress tolerance in plants.

The CBF transcription factors have been widely identified and isolated from rice, tomato, *Brassica napus*, wheat, barley, and maize, which show that the CBF family is large in scale and complex in structure (Yu et al., 2015; Rihan et al., 2017). It has been reported that the *AtCBF* gene was overexpressed in *Brassica napus* transgenic plants and improved the cold tolerance (Huang et al., 2011; Rihan et al., 2017). The combination of the *AtDREB1A* gene with the stress-induced *RD29A* promoter improved the tolerance in transgenic tobacco to drought and cold stress (Bhatnagar-Mathur et al., 2008; Lata et al., 2011). Through phylogenetic analysis of Arabidopsis CBFs and their orthologous genes in other plants, it was found that CBFs were highly conservative in the phylogeny (Shi et al., 2018). In understanding cold stress response among the cotton genotypes, Cai et al. (2019) found that the cotton germplasms of the D genome respond differently to drought stress, in which *Gossypium thurberi* exhibit higher levels of cold stress tolerance by mobilizing the antioxidant enzymes and induction of stress-responsive genes, such as the *CBF4* and *ICE2*. As an important oil and fiber crop, cotton has been planted in more than 70 countries and plays an important role in the global economy. However, cotton yield is often adversely affected by abiotic stresses. Therefore, studying the molecular adaptation mechanism to stress resistance in cotton is of great significance for improving cotton yield. The 21 *CBF* genes have been cloned from *G. hirsutum*, which provides useful clues for understanding the cold tolerance mechanism in cotton. However, due to the limited genome sequence, the expression profile of the *CBF* family, and its phylogenetic relationship with other plants, the role of the *CBF* members is still unclear. In order to better understand the function and evolutionary relationship of the *CBF* gene family in cotton, we analyzed the structural variation and the evolution pattern of the *CBF* family based on the genome-wide data of several cotton species and explored the molecular mechanism of cold adaptation formation in *G. thurberi*. This study provides fundamental information and reference for further research on the molecular mechanism of the *CBF* genes and their regulatory role in cold adaptation in cotton.

## Materials and Methods

### Identification of CBF Family Genes in the Cotton Genomes

Wild diploid cotton species genome data were retrieved from CottonGen^1^ to construct a local BLAST database^2^. The Arabidopsis CBF protein sequences were used as probes to compare with the wild diploid cotton. The *E*-value threshold for BLASTP was set at 1e^–10^ to obtain the final dataset of the CBF proteins. The Pfam (Finn et al., 2014) SMART^3^ databases were employed to confirm each predicted CBF protein sequence (Letunic et al., 2015). Redundant sequences and incomplete sequences were removed. The sequences of 10 *Gossypioides kirkii* and nine *Theobroma cacao* CBF proteins were obtained from the CottonGen (see text footnote 1) and the national center for biotechnology information (NCBI)^4^ databases, respectively. In addition, physicochemical parameters, including the molecular weight (MW) and isoelectric point (*pI*) of each gene product, were calculated using compute the pI/Mw tool from ExPASy^5^, as previously used by Magwanga et al. (2018b) in the evaluation of the physiochemical properties of the *LEA* genes in cotton.

### Sequence Alignment and Phylogenetic Analysis of the Cotton *CBF* Gene Family

An alignment of multiple CBF protein sequences from *A. thaliana*, *Gossypioides kirkii*, and *Theobroma cacao* was generated using the ClustalW program^6^. A neighbor-joining (NJ) analysis of the generated alignment was performed using the unweighted pair-group method with an arithmetic mean algorithm to construct an unrooted phylogenetic tree. Bootstrap value was 1,000, and other parameters were used by default value. The tree was visualized with MEGA 7.0 software (Kumar et al., 2016).

### Gene Structure and C-Terminal Conserved Motifs Analysis

Structural information for the *CBF* genes, including the chromosomal location and gene length, was determined. The exons and introns were predicted by comparing the coding sequences with genomic sequences. The conserved motif analysis of the CBF protein sequences was predicted by MEME online software^7^. Moreover, the Conserved Domains Database (CDD)^8^ was employed to search for the conserved domain information of the CBF and used the TBtools mapping tool to draw the conserved domains (Chen et al., 2020).

### Retrieval and Analysis of Promoter Sequences

The 2,000-bp sequence upstream of ATG was extracted from the transcription start site of the *CBF* gene sequence and submitted the obtained sequence to the PlantCARE website^9^. Identification of possible *cis*-acting elements in the promoter region is used to identify putative *cis*-regulatory elements in the promoter sequence (Higo et al., 1999). In addition, we carried out the subcellular localization prediction of all the CBF proteins by an online tool WoLF PSORT^10^ (Horton et al., 2007).

### RNA Extraction and qRT-PCR Analysis

At three leaf stages, cold stress was imposed by subjecting to cold stress by transferring the seedlings and kept at 4°C under normal light conditions. The leaves were then harvested for RNA extraction at 0, 0.5, 3, 6, 12, and 24 h of post-stress exposure. The total RNA was extracted using the EASYspin plus plant RNA kit (Aidlab Biotech, Beijing, China), following the instructions of the manufacturer. The quality and the concentration of each RNA sample were determined by NanoDrop 2000 spectrophotometer and RNA that fulfilled the standard at 260/280 in a range of 1.80–2.1 was used for further analysis. The primers used for qRT-PCR were designed using primer premier 5 software for all genes (Supplementary Table 3). The cotton *GhActin* gene, (forward primer sequence 5′ATCCTCCGTCTTGACCTTG3′, and reverse primer sequence 5′TGTCCGTCAGGCAACTCAT3′) were used as a reference gene for the analysis. Real-time PCR reactions were carried out in a final volume of 25 μl, using an SYBR Green master mix and an ABI 7500 thermal cycler (Applied Biosystems, Foster City, CA, United States), following the instructions of the manufacturer. The single stranded cDNA was synthesized from reverse transcriptase with TransScript-All-in-One First-Strand cDNA Synthesis SuperMix (TransGen Biotech kit, Beijing, China) for RT-qPCR in harmony with the protocol of the manufacturer. NCBI primer blast was used to design 27 LHC gene primers. The primers details are given in Supplementary Table 1. The 7500 fast real-time system was used for analysis, and 10 μl of Time FastStart Universal SYBR Green Master sigma (ROX) solution (Roche Diagnostics, Mannheim, Germany) per sample was used for RT-qPCR. Total reaction mixture volume was 20 μl, containing 2-μl cDNA, 6-μl RNA free water, 10-μl green SYBR, 1 μl of each forward and reverse primer. Ghactin7 used as a control (Artico et al., 2010). The RT-qPCR was completed with three technical repeats. The expression level of genes was calculated using the formula *E* = 2^–ΔΔCt^.

### Plant Material

The seeds of *Gossypium herbaceum, G. thurberi*, and *Gossypium australe* were pre-germinated in the sand at 25°C for 4 days. Then, seedlings were then transferred to the hydroponic facility equipped with Hoagland nutrient solution (Hoagland and Arnon, 1950). The greenhouse conditions were set at 28°C during the day/25°C at night, the photoperiod was 16 h, and the relative humidity was 60–70%. At the three-leaf stage, cotton seedlings were subjected to cold stress by transferring the seedlings and kept at 4°C under normal light conditions. The leaves were then harvested for RNA extraction at 0, 0.5, 3, 6, 12, and 24 h of post-stress exposure. Each treatment was repeated three times. The leaf samples were put in liquid nitrogen, frozen, and stored at −80°C, awaiting RNA extraction.

### *GthCBF4* Subcellular Localization Analysis

The *Agrobacterium tumefaciens* strain GthCBF4 vector was used for transformation studies. The design of GFP fusion constructs for pCAMBIA2300-eGFP (control GFP). The total RNA (1 μg) isolated from leaves of *G. thurberi* was used for amplification of the first strand of cDNA (TransGen Biotech, Beijing). Specific primers were used to amplify C-repeat-binding factors protein (GthCBF4) gene products from *G. thurberi*. The oligonucleotides used for amplification of GthCBF4 were forward primer 5′GAGAACACGGGGGACTCTAGAATGGTTGATTCTGGGT CGGTTTCT3′ and reverse primer 5′ACCCATGTTAA TTAAGGATCCAATAGAATAACTCCATAAAGG3′ (Sigma, Henan, China). The underlined sequences (TCT AGA and GGA TCC) in the primer pair represent restriction sites for *Xba*I and *Bam*HI, respectively. The reverse primers used for amplification of GthCBF4 genes were designed in a fashion so as to eliminate stop codons of these genes. The PCR-amplified products of GthCBF4 were digested and cloned at the restriction site(s), *Xba*I, and *Bam*HI, respectively, in the vector (GFP) backbone. The PCR cycling conditions were as follows: Step (1) 95°C-3 min, (2) 95°C-15 s, (3) 58°C-15 s, (4) 72°C-1 min, (5) a cycle to Steps 2 to 4 for 35 more times, (6) 72°C-5 min, (7) incubation at 4°C. The PCR was performed using Phanta Max Super-Fidelity DNA polymerase (Vazyme, Nanjing, China). To explore the subcellular localization of the *GthCBF4* gene, a pCAMBIA2300-eGFP-Flag-*GthCBF4* fusion vector of CBF and GFP was transiently infused in the epidermal cells of tobacco leaves; the construct was driven by the 35-s promoter and transformed Agrobacterium LBA4404 competent cells. In addition, Agrobacterium competent cells expressing only the *GFP* gene were used as a negative control. Four-week-old tobaccos cotyledon flat leaves were selected for infusion and cultivated in dark for 24–36 h after infiltration, and then fluorescence observation was performed under a laser confocal microscope.

### Cloning of *GthCBF4*, a CBF Novel Gene in Arabidopsis

To explore the function of the *GthCBF4* gene in Arabidopsis, a constructed pBI121-*GthCBF4* recombinant vector, transformed into the promoter cells of *A. tumefaciens* GV3101 (Reyes et al., 2020), as previously applied by Magwanga et al. (2018a), by adopting a freeze-thaw method (Dupadahalli, 2014), was performed. The wild-type (WT) *A. thaliana* was transformed by adopting the dipping method (Clough and Bent, 1999) by using a 50-mg/ml kanamycin for positive selection up to the third generation (T3). At the second generation, also referred to as the T2 generation, the GthCBF4 overexpressed lines were identified through a polymerase chain reaction; two high-overexpressed transgenic lines were obtained, and grown to generate the stable T3 generation.

### Phenotypic Identification, Physiological and Biochemical Parameters of Wild-Type and GthCBF4 Overexpressed Plants Under Cold Stress

The transgenic and wild-type *A. thaliana* was grown in plastic bowls for 2 weeks; the growth pattern between the transgenic and the wild types was not significantly different. The two types, transgenic and wild type Arabidopsis, were placed in an environment of −15°C for 3 h. After the treatment, the treated plants were moved to a 4°C-light incubator to thaw for 4 h, and finally, they were cultivated under normal light conditions at 23°C. After 7 days, photographs were taken to aid the counting of the survival rate of the plants. The germination rate and the root length were determined under cold stress; a *t*-test was used to verify the significance of the difference in root length between the mutant and the wild types. The trypan blue staining method (Berrocal-lobo, 2016) was adopted to reflect the cell damage under cold stress. Moreover, the DAB staining was applied to reflect the accumulation of peroxidase under cold stress. DAB staining was performed using a DAB chromogenic kit (Nanjing Jiancheng Bioengineering Institute, Nanjing, China).

### Statistical Analysis

The experiments were done in three biological replicates, and the data were statistically analyzed by ANOVA procedure (Molugaram and Rao, 2017), using statistical analysis software (SAS) version 9.3. The least significant difference test (*p* ≤ 0.05) was used for the mean comparison.

## Results

### Identification of the *CBF* Gene Families in the Cotton Genomes

The availability of the whole sequences for the seven cotton species enabled us to identify the CBF proteins harbored in their genome. The protein family (Pfam) domain PF00847 was used as the query to obtain the CBF proteins. Finally, 29 members of the *CBF* genes were identified in *G. herbaceum*, 28 members in *Gossypium arboreum*, 25 members in *G. thurberi*, 21 members in *Gossypium raimondii*, and 30 members in *Gossypium turneri*, and *Gossypium longicalyx* had 26 members. In contrast, *G. australe* had 15 *CBF* genes. Three representative cotton species from these seven species were then chosen for further detailed analysis: *G. herbaceum* (29), *G. thurberi* (25), and *G. australe* (26). The CBF-coding sequences (CDS) length in *G. herbaceum* ranged from 306 to 1,230 bp, while, for *G. australe*, the CDS ranged from 429 to 1,077 bp. In the analysis of the physicochemical properties of the CBF proteins, the results showed a great variation; for instance, the CBF proteins obtained from the *G. herbaceum*, their molecular weights (MW) ranged from 11.24188 to 39.02073 kDa; isoelectric value (*pI*) ranged from 5.26 to 10.27, while, in *G. thurberi*, the MW of the proteins encoded by the *CBF* genes ranged from 16.17923 to 45.59153 kDa; the *pI* ranged from 5.11 to 10.83. Similarly, in *G. australe*, it ranged from 15.67636 to 40.86796 kDa. The *CBF* genes differed substantially by the encoded protein size and its biophysical properties (Supplementary Table 1).

### Phylogenetic Analysis of the Cotton *CBF* Gene Family

**FIGURE 1:**
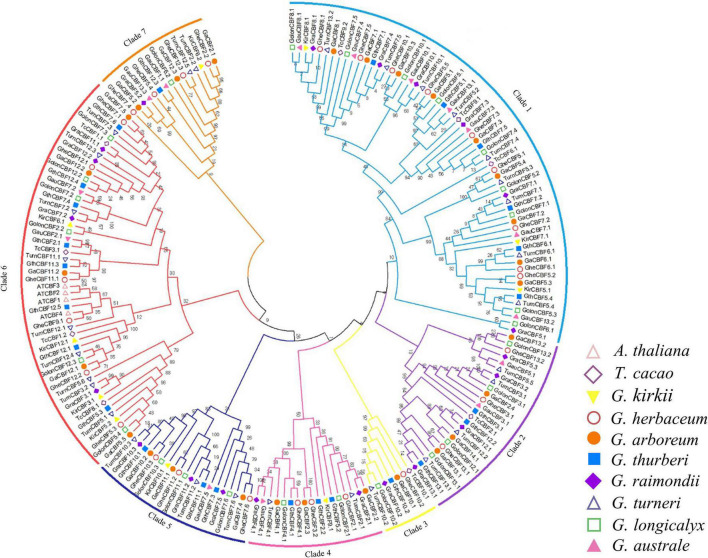
Phylogenetic tree analysis of C-Repeat Binding Factor (CBF) transcription factors. Protein sequences alignment was done by ClustalX, the phylogenetic tree built by MEG 7.0 software using 1000 bootstrap replication *via* a neighbor-joining approach. Different colors were used to differentiate the homologous clusters.

To determine the phylogenetic relationship of the CBF proteins, we constructed a phylogenetic tree by MEGA 7.0, using the neighbor-joining (N.J.) method with minimal evolution and maximum parsimony. The CBF proteins were clustered into seven clades and designated as Clades 1 to 7 (Figure 1). Clade 1 contained 61 CBF protein sequences, while Clade 3 contains only seven CBF amino acid sequences. Consistent with the previous classification, all Arabidopsis CBFs were distributed among the Clade 6 (Haake, 2002; Huang et al., 2011; Rihan et al., 2017). Except *G. australe* did not appear in Clade 3, the other cotton species were distributed in all seven groups.

### Chromosomal Mapping, Gene Structure, and C-Terminal-Conserved Motif Analysis

**FIGURE 2:**
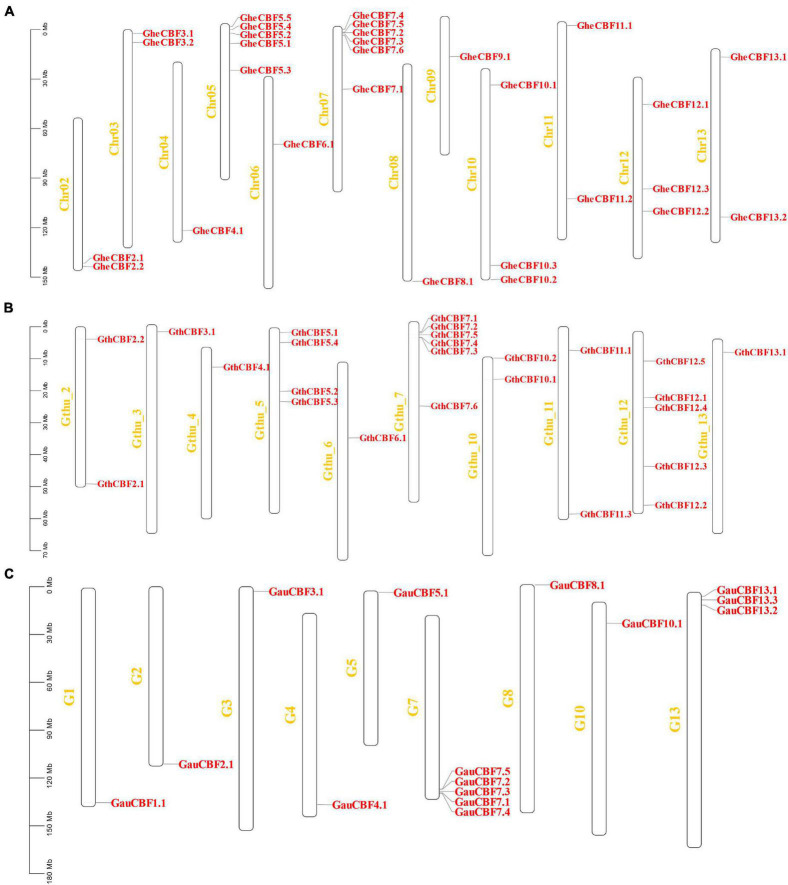
Chromosomal positions of the *CBF* genes. **(A)** Chromosomes of *G. herbaceum*, **(B)** chromosomes of *G. thurberi*, and **(C)** chromosomes of *G. australe*.

All the genes located on various chromosomes in the three cotton genomes were named according to their positions on the chromosome. In *G. herbaceum*, a member of the diploid type of the A genome, the *CBF* genes were mapped on all the chromosomes, except chromosome Chr01, which harbored no *CBF* genes. The highest gene loci were observed in chromosome Chr05 and Chr07 with five and six genes, respectively, while the lowest gene loci were observed in chromosome Chr04, 06, 08, and Chro09 with a single gene locus in each (Figure 2A). In the diploid of the D genome, *G. thurberi*, chromosome Gthu_1, Gthu_8, and Gthu_9 harbored no genes, while chromosomes, Gthu_5, Gthu_7, and Gthu_12 had more gene loci. However, the highest gene loci were noted in chromosome Gthu_05, 07, and 12 with four, six, and five *CBF* genes, respectively; similarly, the lowest gene loci were noted on chromosome Gthu_3, 6, and 13 with a single gene locus each (Figure 2B). Finally, in *G. australe* diploid cotton of the G genome, no *CBF* genes were found in chromosomes G6, G9, G11, and G12, but the highest gene loci were only observed in chromosome G7 with five genes (Figure 2C).

**FIGURE 3:**
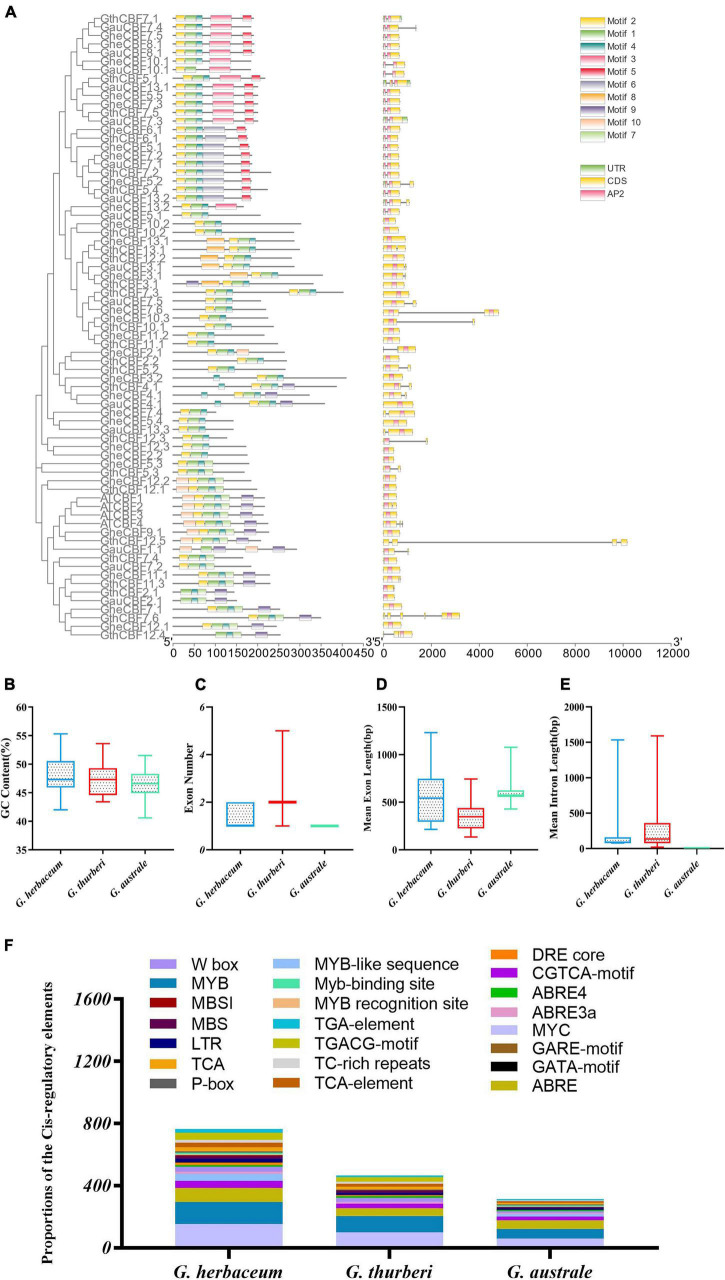
Conserved domain and gene structure analysis of CBF family protein sequences from the evolutionary level in cotton. Different colors denote different motifs. **(A)** Physiochemical properties and *cis-*regulatory element analysis of the proteins encoded by the cotton *CBF* genes. **(B–E)** Guanine-cytosine (G.C.) content, exon number, mean exon length, and mean intron length. **(F)** *Cis-*regulatory elements obtained for the various proteins encoded by the *CBF* genes in the *G. herbaceum*, *G. thurberi*, and *G. australe.*

In order to analyze the motifs, we employed MEME to detect conserved motifs in the *CBF* family. There were 10 conserved motifs distributed in each CBF family (Figure 3A). Almost all CBF proteins had the same number of motifs; three motifs were prominent among the CBF proteins, motifs 1, 2, and 4. Analyzing the arrangement of exons and introns can provide important insights into the evolution of gene families (Colebrook et al., 2014). To study the exon/intron structure of the *CBF* gene, the CDS and the genome sequence were compared. The results showed that most of *G. herbaceum* and *G. australe* harbored no intron, and indication that the genes were highly conserved, but, for the *G. thurberi*, the least number of exons was two, but others contained a higher number (Figures 3B–E). Basically, the members of the same group phylogenetically harbored a similar number of the intron-exon ratio. Stress regulates the expression of primary and specialized metabolism genes at the transcriptional level *via* transcription factors binding to specific *cis*-elements; a number of the *cis-*regulatory elements were found to be associated with the various cotton *CBF* genes, for instance, MYB, ABA-responsive element (ABRE), and long-term repeat (LTR), among others (Figure 3F); the MYB and ABRE are among the top-ranked stress responsive *cis-*regulatory elements (Ambawat et al., 2013). The detection of a myriad of *cis-*regulatory elements reveals the significant role played by the members of the cotton *CBF* genes in enhancing stress tolerance, more so cold stress in plants.

An online tool wolf sport was employed to determine the possible cellular sublocalization of the proteins encoded by *CBF* genes. Among the three cotton species, the highest proportions of CBF proteins were embedded in the nucleus. Moreover, the nucleus is the central regulator of cellular activities. However, other signals were observed in different cellular compartments, such as the chloroplast, mitochondria, plasma membrane, vacuole membrane, and chloroplast (Supplementary Table 2).

### RNA-Seq Analysis and RT-qPCR Validation of the *CBF* Genes Under Cold Stress Conditions

**FIGURE 4:**
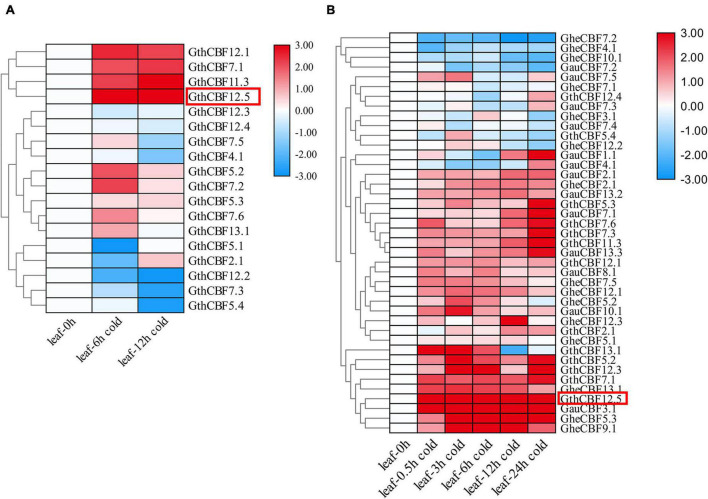
RT-qPCR validations of transcript levels evaluated by RNA-Seq in *G. thurberi* under cold stress. **(A)** Heat map depicting log2 (fold change) of the transcription factor. **(B)** Heat map log2 (fold change) of the *CBF* genes expression under cold stress conditions. The samples were collected at 0, 0.5, 3, 6, 12, and 24 h for leaf tissues. Red: upregulated genes; blue: downregulated; black: none-expressed genes. Red Box denotes the key gene.

*Gossypium thurberi* transcriptome data were used to analyze the expression patterns of the 25 *CBF* genes under cold stress; however, out of the 25 identified *CBF* genes, only 17 exhibited differential expression, while the other eight showed no expression, and thus, we excluded from further analysis (Figure 4A). Out of the 17 differentially expressed genes under cold stress as per the RNA-seq^11^, 12 genes were selected as per their expression patterns, upward, downward, and partially expressed genes; the 12 *CBF* genes exhibited differential expression; moreover, 15 and 13 *CBF* genes from *G. herbaceum* and *G. australe*, respectively, were also profiled through RT-qPCR (Figure 4B). Among the 12 genes in *G. thurberi*, 10 genes exhibited significantly higher upregulation, with only two genes being downregulated. The expression trend was consistent with the transcriptome data. In *G. herbaceum*, nine genes were upregulated, and six were downregulated. In *G. australe*, eight were upregulated and five were downregulated. But, more significantly, out of all the *CBF* genes profiled, GauCBF3.1 and GthCBF12.5 were most highly upregulated in the leaf across the periods in which the plants were subjected to cold stress. The leaf plays an important role in photosynthesis, and the stomata are the key to this function, for it controls both photosynthesis and respiration processes (Hajihashemi et al., 2018). Moreover, the process of photosynthesis involves the mechanisms of capturing light energy, which is then transformed to carbohydrates; this whole process is susceptible to low temperature (Stewart et al., 2016). The higher level of upregulation among the *CBF* genes across the various cotton types indicates that the proteins encoded by the *CBF* genes could play an integral role in enhancing cold acclimatization in cotton plants. Moreover, by integrating the transcriptome data and RT-qPCR result, we selected one of the highly upregulated *CBF* genes, *GthCBF12.5* (*GthCBF4*), for further validation.

### Experimental Validation of Subcellular Localization of Cotton CBF Proteins

**FIGURE 5:**
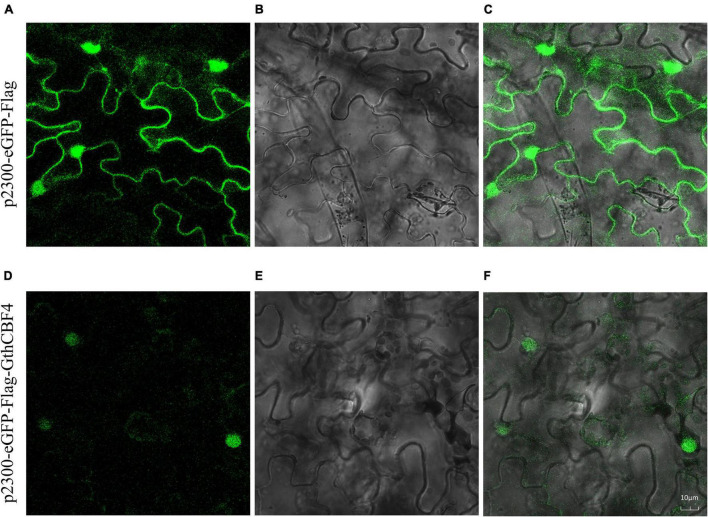
Localization of pCAMBIA2300-eGFP-Flag-*GthCBF4* in tobacco leaf. **(A–C)** Tobacco epidermal cells transformed with pCAMBIA2300-eGFP; **(D–F)** Onion epidermal cells transformed with pCAMBIA2300-eGFP-Flag-*GthCBF4*. **(A,D)** A light field with the magnification of X400 to display morphology. **(B,E)** Dark field images for the detection of green fluorescent protein (GFP) fluorescence. **(C,F)** Superimposed light and dark field images.

The results showed that none of the pCAMBIA2300-eGFP-Flag-*GthCBF4*-infused plants showed green fluorescence signals on both the nucleus and cell membrane. In contrast, the fusion protein pCAMBIA2300-eGFP-Flag-*GthCBF4* only had green fluorescence signals in the nucleus (Figure 5), indicating that the protein encoded by the gene was localized in the nucleus. The results were in agreement with the bioinformatics prediction of the possible cell compartmentalization of the CBF proteins. The presence of these proteins encoded by the *CBF* genes could possibly indicate that these genes have a critical role under cold stress conditions; moreover, posttranscriptional regulation at the pre-mRNA processing and export from the nucleus plays a role in cold acclimation (Chinnusamy et al., 2010).

### Phenotype and Biochemical Evaluation of *GthCBF4*-Overexpressed Arabidopsis Plants Under Low Temperature

**FIGURE 6:**
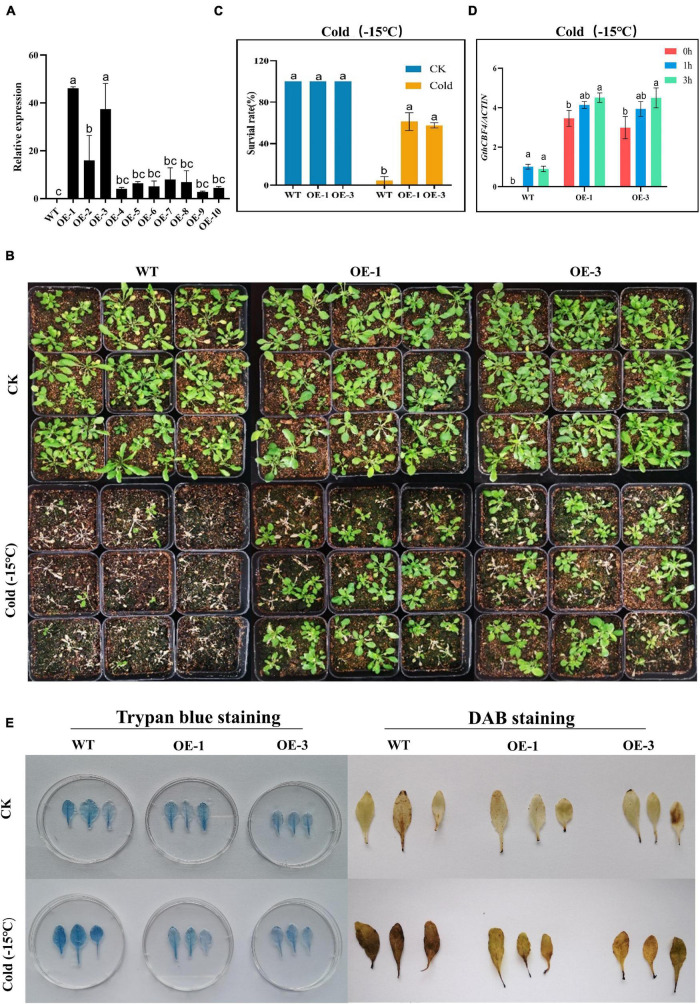
Phenotype identification and cell damage identification of GthCBF4-overexpressed Arabidopsis under low temperature. **(A)** The quantitative expression level of *GthCBF4* gene in transgenic Arabidopsis. **(B)** –15°C treatment restores the growth status of wild-type and transgenic *Arabidopsis thaliana* after culture. **(C)** Freezing survival assay. **(D)** Log10 (fold change) of the *GthCBF4* genes expression in –15°C treatment. **(E)** Trypan blue staining and DAB staining. The letters a/b indicate statistically significant differences (two-tailed, *p* < 0.05) between the samples in each treatment.

Two highly overexpressed (OE) lines OE-1 and OE-3 were selected for phenotypic and biochemical evaluation (Figure 6A); phenotypically, the GthCBF4-overexpressed lines (OE-1 and OE-3) showed no significant difference with the wild types under normal conditions, but, when the plants were exposed to cold stress, the survival of the WT was significantly reduced, while the OE-1 and OE-3 showed a higher level of survival (Figure 6B), in which the OE plants survival rate was estimated at 60% compared to the WT with only a 2% survival rate (Figure 6C). Similar results were obtained by Tang et al. (2019) in which the survival rate among the overexpressed lines was estimated at 64.9%, 62.2%. In determining the expression levels of the *GthCBF4*-overexpressed gene, at 0 h, the WT showed no expression, but, at 1 h and 3 h of cold-stress exposure, the *CBF* genes were partially inducted but insignificantly, slightly below one, while the reverse was observed among the OE plants. The expression levels of the GthCBF4- overexpressed gene were significantly high, with 0 h showing expression levels close to 3-fold, while, at 1 h and 3 h, the expression levels of the GthCBF4-overexpressed gene were above 4-fold (Figure 6D). Under normal conditions, the stained blue area on the leaves of the transgenic overexpression plants and wild-type plants was minimal. While under cold treatment, the blue regions on transgenic leaves were significantly smaller than the wild type; the color depth was also lighter. Furthermore, the DAB-staining method was used to reflect the accumulation of H_2_O_2_ in Arabidopsis leaves. The accumulation of H_2_O_2_ in the transgenic overexpression leaves and the wild-type was very low under normal conditions, and the DAB-staining region was hardly seen. But, after cold treatment, the DAB staining area on the wild-type Arabidopsis leaves was more pronounced compared to the stained region in the wild type, and the color depth was also deeper (Figure 6E). Knockdown of the *OsGRXS17* gene in rice significantly increased the H_2_O_2_ accumulation. The gene plays a critical role in the induction of ABA. ABA-induced accumulation of H_2_O_2_, which is synthesized in guard cells, is essential for stomatal closure by activating plasma membrane Ca^2+^ channels. In the absence of ABA, the leaves of mutant plants exhibited slightly higher H_2_O_2_ levels on the basis of DAB staining than the wild-type plants (Hu et al., 2017). The results showed that the overexpression of the *CBF* gene in the transgenic lines improved the ability of the plants to oxidize the oxidative agents such as the H_2_O_2_, thereby reducing the levels of oxidative damage in the plants.

### Evaluation of Physio-Morphological Traits in *GthCBF4*-Overexpressed and Wild-Type Plants Under the Low-Temperature Environment

**FIGURE 7:**
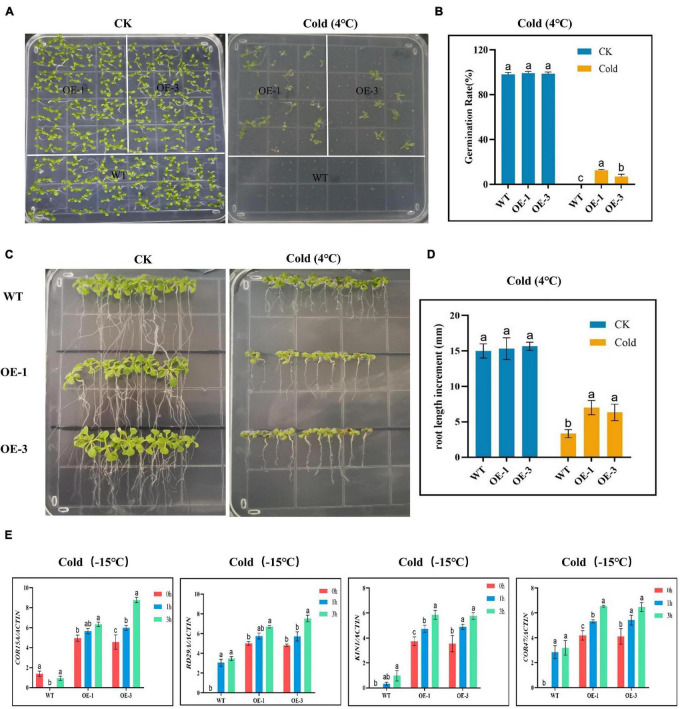
Determination of the growth phenotypes and regulatory gene expression of transgenic Arabidopsis under cold stress conditions. **(A,B)** Germination rate determination. **(C,D)** Root length determination. **(E)** The expression levels of abiotic stress-responsive gene. The letters a/b indicate statistically significant differences (two-tailed, *p* < 0.05) between the samples in each treatment.

Germination of the OE and WT lines showed no significant differences under normal or controlled conditions; however, under cold stress, none of the WT germinated, while the OE lines showed some level of germination (Figures 7A,B), an indication that the OE lines were significantly improved and were able to adapt to cold stress condition. In the evaluation of the root lengths, no significant differences were observed under controlled conditions, but, under cold stress conditions, the OE lines showed higher root lengths compared to the WT (Figures 7C,D). Thus, the overexpression of the *CBF* genes could be playing a role either in the rate of cell division and or cell elongation. We further evaluated known stress-responsive genes, such as the *COR15A*, *RD29A*, *KIN1*, and *COR47* (Zhao et al., 2016). The OE lines significantly showed higher expression levels to all the stress-responsive genes profiled (Figure 7E). The high induction levels of the stress-responsive genes in the OE lines showed that the overexpressed genes do not suppress the expression of the stress-responsive genes, but do promote their expression, an indication that the overexpressed gene could be playing a vital role in enhancing cold stress tolerance in plants.

## Discussion

Cotton is an important economic crop, which supports the economies of several countries globally; moreover, it is the primary source of natural fiber critical raw material for the textile industries (Reddy and Yang, 2009); however, in the recent past, its global production has significantly shrunken due to climate change. Rainfall has become erratic and scarce. Even the average daily temperature has increased dramatically with prolonged periods of cold weather, thereby affecting the growth and development of the plant. To improve the performance of current elite cultivars, a number of strategies have been proposed; among them is the utilization of the transcription factors and other novel genes of the plants to improve the adaptability of the current cotton germplasms to ever-changing environmental conditions. Cold stress has been a major concern in cotton production, but the climate dynamics have increased the deleterious effects of cold stress. For instance, in the 1980s, the United States loss due to the chilling impacts was estimated at 60 million dollars (Chaurasiya and Singh, 2019). Furthermore, cold stress causes considerable agricultural yield loss in very sensitive crops, particularly in sensitive crops like maize, rice, and chickpea (Thakur and Kumar, 2010). To increase the chance of survival, plants have adopted numerous mechanisms to cope with the ever-changing environmental conditions, one of which is the evolution of novel genes with tremendous effects on improving the adaptability of plants to various environmental stress factors, for instance, the *LEA* genes have been found to offer drought-stress tolerance in cotton (Magwanga et al., 2018a).

Although abiotic stress is a significant challenge in cotton growth, there is no detailed study on the *CBF* gene in cotton (Bo et al., 2007). In previous studies, the *CBF* family has been identified in cotton (*Gossypium hirsutum*) (Ma et al., 2014), wheat (Mohseni et al., 2012), lettuce (Sunchung et al., 2020), *Brassica napus* (Lv et al., 2020), Barley (Marquez-cedillo et al., 2005), and soybean (Kidokoro et al., 2015), but there are few studies on diploid cotton. In this work, genome-wide identification, characterization, and functional analysis of the proteins encoded by the cotton *CBF* genes were done, in which 29, 25, and 15 CBF proteins encoded by the *CBF* genes were identified in *G. herbaceum*, *G. thurberi*, and *G. australe*, respectively. The results obtained showed that the proteins encoded by the *CBF* genes in cotton were higher compared to other plants, such as lettuce with 14, *Brassica napus* with 10, and soybean with 14 *CBF* genes, but less than hexaploid wheat with 65 *CBF* genes and barley with 20 *CBF* genes. The high number of the proteins encoded by the *CBF* genes in cotton perhaps could explain the significant role they play in plants in relation to stress tolerance. Moreover, studies have found that Arabidopsis CBF family members have an AP2 domain, and each has a conserved amino acid sequence upstream and downstream of AP2. The upstream of AP2 is PKK/PKKPAGR (RAGRxxKFxETRHP), and the downstream is DSAWR (Okamuro et al., 1997). If the PKK/PKKPAGR mutation can inhibit the binding ability of CBF to the COR promoter of its downstream genes, thereby weakening the level of CBF regulation, this sequence is necessary for CBF to perform its transcription factor function. The cotton genome contains a large and complex CBF subfamily, with conserved AP2/EREBP domains and with CBF-like characteristics, indicating that cotton CBFs have a similar function with other CBFs in dicotyledons (Siddiqua and Nassuth, 2011). Phylogenetic analysis showed that the *CBF* families are divided into seven groups; among these genes, *CBF1*, *CBF2*, and *CBF3* are all induced by cold stress in *Arabidopsis thaliana*. Therefore, we speculate that *CBF* genes may also respond to abiotic stress, especially to cold. Further analysis showed that the isolated *CBF* gene was highly expressed under cold stress, consistent with previous research results.

The cotton *CBF* genes phylogenetic analysis revealed that the entire members of the cotton CBF family were grouped into seven clades; this deviates from the previous findings in which five clusters were observed among the CBF members obtained from lettuce (Sunchung et al., 2020) but were in agreement with findings obtained for the Tea Plant (*Camellia sinensis*) in which the CBF and its homologs were classified into six groups (Hu et al., 2020). In plants, the transcriptional regulation of osmotic stress response mainly depends on two main *cis-*regulatory elements related to stress response genes ABREs and dehydration response elements (DREs). The two *cis-*regulatory aspects were found to be associated with the *CBF* genes, which are indicators that the members of the *CBF* gene family could, perhaps, be playing a significant role in enhancing cold stress tolerance in plants. Moreover, the DREs are mainly involved in ABA-independent pathways (Liu S. et al., 2018), and ABRE is responsible for detecting ABA-mediated osmotic stress signals (Kim et al., 2011). In this study, the *CBF* genes detected for the three cotton species were rich in stress-response genes (ABRE), dehydration response element (DRE), and cold stress response element (LTR). Moreover, the C-repeat-binding factors (CBFs) are pivotal signaling genes, which are rapidly induced by cold and bond to the C-repeat/dehydration-responsive motif in the promoter region, more so in the downstream cold-responsive genes, with an important role in enhancing cold stress tolerance in plants. In the identification of the *CBF* genes in tea, a number of the identified genes were found to be associated with various stress responsive promoters, more importantly the CRT/DRE motif (Wang et al., 2019). It is suggested that exogenous environmental stress can induce the expression of the *CBF* gene through its response to *cis-*regulatory elements and further improve the resistance of plants to environmental stress.

It is universally accepted that transcription factors must be present in the nucleus to perform their functions (Wang et al., 2008). In evaluating the possible cell compartmentalization of the proteins encoded by the *CBF* genes, both prediction and experimental evaluation showed that most of the CBF proteins are localized within the nucleus. Several studies have found that a number of stress-responsive proteins are sub-localized within the nucleus, for instance, calmodulin-regulated proteins; in plants, the activity of a pea apyrase, PsNTP9, is localized in the nucleus, and has a significant role in enhancing resistance to heavy metals and xenobiotic compounds (Virdi et al., 2015). Moreover, when plants are exposed to environmental stress, there is increased release of oxidizing agents, which destroys the DNA to counteract the DNA damage; plant cells evolved mechanisms for the DNA repair in both nucleus and mitochondria (Gruszka, 2015). Thus, the abundance of the CBF proteins in the nucleus could be significant in DNA repair as a result of oxidative damage.

Overexpression of Arabidopsis *CBF* gene in other plant species or overexpression of *CBF* of other species in Arabidopsis has revealed the potential of the *CBF* genes in enhancing frost resistance (Tondelli et al., 2011). Moreover, it has been shown that the *CBF* gene plays an important role in plant cold acclimatization; downregulation of *CBF1* and *CBF3* results in a 25–50% reduction in tolerance to cold stress levels in pre-cold treated mutant plants (Miura and Furumoto, 2013). In this study, the *GthCBF4* was strongly upregulated in cotton seedlings under cold treatment. The survival rate of *GthCBF4* transgenic *Arabidopsis thaliana* plants was significantly improved after freezing treatment. Furthermore, the inducer of CBF Expression (ICE) is a pioneer of cold acclimation, an MYC-type basic helix-loop-helix family transcription factor (TF; Zarka et al., 2003). It has been reported that ICE1 plays a significant role in C-repeat binding factor 3 (CBF3) cold induction (Chinnusamy et al., 2003). When plants encounter cold stress, ICE1 could be mobilized from JAZs bound by DELLAs and trigger the expression of CBF3. The CBF3 activates the expression of GA2ox7 to reduce the bioactive gibberellic acid (GA) level, which enhances the accumulation of DELLAs. Therefore, DELLAs can control the cold induction of CBF3 through ICE1 *via* JAZs. Moreover, the trypan blue staining, DAB staining, and the GthCBF4-overexpressed plants significantly reduced cold injury compared to the wild types, a strong indicator of the possible roles of *CBF* genes in enhancing cold-stress tolerance in plants. Moreover, root growth was significantly higher in GthCBF4-overexpressed plants. The trypan blue staining is critical to determining the level of cell death. When plants are exposed to various forms of stress factors, the cells undergo a series of effects, including cell damage, and eventually, cell death, depending on the intensity or tolerance nature of the plant. The overexpressed plants exhibit significantly low staining as compared to the wild types. The dye only enters into dead cells and not living ones; thus, the high blue color intensity in wild types showed that the level of cell death was significantly higher due to cold-stress effect (Zhang et al., 2017).

To further elucidate the critical roles played by the proteins encoded by the *CBF* genes, known stress-responsive genes were profiled, and the results showed that overexpression of the *CBF* gene significantly increased the induction levels of the stress-responsive genes. In general, the expression level of the four *COR* genes was significantly correlated with the freezing tolerance level. Furthermore, *COR15A*, which encodes the chloroplast-targeted polypeptide, enhances the cold resistance of the chloroplasts (Thalhammer et al., 2014). In addition, the *CBF* genes can induce the expression of the *COR47*, *RD29A*, and *KIN1* genes, thereby improving the tolerance level of the plant to cold stress (Zhao et al., 2016). The upregulation of the stress-responsive genes further confirmed that the ability of GthCBF4-overexpressed plants to tolerate the effects of cold was significantly higher compared to the wild types. In the past two decades, the transcriptional network of CBF-signaling pathways has been extensively studied, and many *COR* genes have been identified in genome-wide expression profiles. About 10–25% of them are regulated by CBF, which implies that more early cold-regulated transcription factors are mainly involved in improving cold tolerance. Moreover, the mutational effect on the DRE motif revealed that the mutant crops were highly susceptible to cold stress as compared to non-mutant (Lei et al., 2014), which implied that mutations in DRE motifs impair the induction of the CORs under cold stress.

## Conclusion

Based on the genome-wide identification and functional characterization of the proteins encoded by the *CBF* genes, a total of 69 *CBF* genes were identified among the three diploid cotton species, in which 29, 25, and 15 CBF proteins were identified in *G. herbaceum*, *G. thurberi*, and *G. australe*, respectively. The RNA data and the expression profiling of the *CBF* genes revealed that higher proportions were upregulated under cold stress conditions. Moreover, overexpression of the *Gthu17439 (GthCBF4)* gene in the model plant, Arabidopsis, revealed that the ability of the GthCBF4-overexpressed plants to tolerate cold stress was higher compared to the wild types, as evident in the germination, root growth, and induction of various stress-responsive genes. The results, therefore, provide fundamental information for future exploration of the *CBF* genes in the development of more robust cotton germplasms, which are highly resilient to cold and or chilling stress.

## Data Availability Statement

The original contributions presented in the study are included in the article/Supplementary Material, further inquiries can be directed to the corresponding author/s.

## Author Contributions

JL, RM, and FL: conceptualization. JL, RM, YX, TW, and FL: methodology, software, formal analysis, investigation, writing – original draft preparation, and visualization. JL, RM, YX, TW, FL, and ZZ: validation. SA, KW, ZZ, XC, and FL: resources, supervision, project administration, and funding acquisition. JL, RM, YX, JK, EO, TW, and FL: data curation. JL, RM, YX, TW, JZ, YH, SA, KW, ZZ, XC, and FL: writing – review and editing. All authors have read and agreed to the published version of the manuscript.

## Conflict of Interest

The authors declare that the research was conducted in the absence of any commercial or financial relationships that could be construed as a potential conflict of interest.

## Publisher’s Note

All claims expressed in this article are solely those of the authors and do not necessarily represent those of their affiliated organizations, or those of the publisher, the editors and the reviewers. Any product that may be evaluated in this article, or claim that may be made by its manufacturer, is not guaranteed or endorsed by the publisher.

## Footnotes

1
https://www.cottongen.org/

2
https://blast.ncbi.nlm.nih.gov/Blast.cgi?PAGE=Proteins

3
http://smart.embl-heidelberg.de/

4
https://blast.ncbi.nlm.nih.gov/Blast.cgi

5
http://www.expasy.org/tools/

6
https://www.genome.jp/tools-bin/clustalw

7
https://meme-suite.org/meme/

8
https://www.ncbi.nlm.nih.gov/cdd/

9
http://www.dna.affrc.go.jp/htdocs/PLACE/

10
http://www.genscript.com/wolf-psort.html

11
https://www.ncbi.nlm.nih.gov/sra/PRJNA554555
