# Supplementary material for: Functional Characterization of Cotton C-Repeat Binding Factor Genes Reveal Their Potential Role in Cold Stress Tolerance
Source: Front Plant Sci. 2021 Dec 8;12:766130. doi: 10.3389/fpls.2021.766130 (PMC8692369; doi:10.3389/fpls.2021.766130)
Supplement: Supplementary file 1 [file Table_1.DOCX]

Table S1. Physiochemical properties of the *CBL* genes, indicating the gene name, GC content, exon number, exon mean length, gene location and the isoelectric point.

| Gene ID | Gene Name | GC Content (%) | Exon Number | Mean Exon Length(bp) | Mean Intron Length(bp) | Protein Length(aa) | Molecular Weight(kDa) | Isoelectric Point | Gene Location | CDS Length(bp) |
| --- | --- | --- | --- | --- | --- | --- | --- | --- | --- | --- |
| Ghe02G18690 | GheCBF2.1 | 44.90% | 1 | 792.0 | No Intron | 263 | 29363.95 | 6.14 | Chr02 | 792 |
| Ghe02G19970 | GheCBF2.2 | 48.70% | 1 | 528.0 | No Intron | 175 | 19434.6 | 9.41 | Chr02 | 528 |
| Ghe03G03390 | GheCBF3.1 | 46.90% | 1 | 1062.0 | No Intron | 353 | 39020.73 | 9.26 | Chr03 | 1062 |
| Ghe03G07060 | GheCBF3.2 | 46.30% | 1 | 1230.0 | No Intron | 409 | 45757.44 | 6.19 | Chr03 | 1230 |
| Ghe04G19140 | GheCBF4.1 | 45.40% | 1 | 969.0 | No Intron | 322 | 36706.04 | 6.01 | Chr04 | 969 |
| Ghe05G12970 | GheCBF5.1 | 47.60% | 2 | 301.5 | 97 | 200 | 20192.55 | 6.38 | Chr05 | 603 |
| Ghe05G06300 | GheCBF5.2 | 51.50% | 2 | 214.5 | 101 | 142 | 21067.96 | 9.03 | Chr05 | 429 |
| Ghe05G27290 | GheCBF5.3 | 46.10% | 1 | 558.0 | No Intron | 185 | 19723.45 | 5.2 | Chr05 | 558 |
| Ghe05G03790 | GheCBF5.4 | 47.30% | 1 | 543.0 | No Intron | 180 | 15746.45 | 7.77 | Chr05 | 543 |
| Ghe05G01760 | GheCBF5.5 | 53.00% | 2 | 270.0 | 82 | 179 | 22280.83 | 7.77 | Chr05 | 540 |
| Ghe06G13000 | GheCBF6.1 | 49.40% | 2 | 261.0 | 79 | 173 | 19586.52 | 9.24 | Chr06 | 522 |
| Ghe07G18320 | GheCBF7.1 | 51.00% | 1 | 306.0 | No Intron | 101 | 27721.94 | 6.97 | Chr07 | 306 |
| Ghe07G03800 | GheCBF7.2 | 47.80% | 2 | 286.5 | 87 | 190 | 21111.88 | 9.01 | Chr07 | 573 |
| Ghe07G04380 | GheCBF7.3 | 46.70% | 2 | 280.5 | 85 | 186 | 22403.13 | 6.97 | Chr07 | 561 |
| Ghe07G01750 | GheCBF7.4 | 45.60% | 2 | 301.5 | 1534 | 200 | 11241.88 | 10.27 | Chr07 | 603 |
| Ghe07G03420 | GheCBF7.5 | 42.00% | 2 | 330.0 | 78 | 219 | 21105.74 | 6.33 | Chr07 | 660 |
| Ghe07G05270 | GheCBF7.6 | 45.70% | 1 | 759.0 | No Intron | 252 | 24597.79 | 9.16 | Chr07 | 759 |
| Ghe08G32310 | GheCBF8.1 | 46.70% | 2 | 288.0 | 82 | 191 | 21148.07 | 8.71 | Chr08 | 576 |
| Ghe09G06510 | GheCBF9.1 | 51.50% | 1 | 681.0 | No Intron | 226 | 24941.93 | 5.26 | Chr09 | 681 |
| Ghe10G08080 | GheCBF10.1 | 46.80% | 2 | 277.5 | 336 | 184 | 20772.59 | 8.83 | Chr10 | 555 |
| Ghe10G32620 | GheCBF10.2 | 50.20% | 1 | 675.0 | No Intron | 224 | 32783.07 | 7.79 | Chr10 | 675 |
| Ghe10G27020 | GheCBF10.3 | 43.10% | 1 | 909.0 | No Intron | 302 | 24201.83 | 5.6 | Chr10 | 909 |
| Ghe11G03020 | GheCBF11.1 | 46.40% | 1 | 687.0 | No Intron | 228 | 25733.47 | 5.53 | Chr11 | 687 |
| Ghe11G31060 | GheCBF11.2 | 42.90% | 1 | 648.0 | No Intron | 215 | 23928.62 | 5.57 | Chr11 | 648 |
| Ghe12G07790 | GheCBF12.1 | 48.40% | 1 | 735.0 | No Intron | 244 | 27058.26 | 8.29 | Chr12 | 735 |
| Ghe12G15310 | GheCBF12.2 | 50.90% | 1 | 519.0 | No Intron | 172 | 20415.82 | 5.6 | Chr12 | 519 |
| Ghe12G12080 | GheCBF12.3 | 51.40% | 1 | 555.0 | No Intron | 184 | 19086.25 | 9.52 | Chr12 | 555 |
| Ghe13G04590 | GheCBF13.1 | 49.60% | 1 | 861.0 | No Intron | 286 | 31688.57 | 6.13 | Chr13 | 861 |
| Ghe13G19950 | GheCBF13.2 | 55.30% | 1 | 501.0 | No Intron | 166 | 18185.22 | 9.03 | Chr13 | 501 |
| Ga02G0234 | GaCBF2.1 | 48.67% | 1 | 528.0 | No Intron | 175 | 19434.6 | 9.41 | Chr02 | 528 |
| Ga02G0354 | GaCBF2.2 | 43.08% | 1 | 1026.0 | No Intron | 341 | 37942.27 | 6.01 | Chr02 | 1026 |
| Ga03G2174 | GaCBF3.1 | 46.55% | 1 | 1218.0 | No Intron | 405 | 45276.97 | 6.32 | Chr03 | 1218 |
| Ga03G2468 | GaCBF3.2 | 47.85% | 1 | 909.0 | No Intron | 302 | 32758.46 | 8.67 | Chr03 | 909 |
| Ga04G0455 | GaCBF4.1 | 45.93% | 1 | 984.0 | No Intron | 327 | 37346.69 | 6.01 | Chr04 | 984 |
| Ga05G0167 | GaCBF5.1 | 47.38% | 3 | 216.0 | 282.5 | 215 | 23977.94 | 7.69 | Chr05 | 648 |
| Ga05G0372 | GaCBF5.2 | 51.05% | 1 | 429.0 | No Intron | 142 | 15762.41 | 6.74 | Chr05 | 429 |
| Ga05G0613 | GaCBF5.3 | 46.24% | 2 | 279.0 | 101 | 185 | 21137.11 | 9.35 | Chr05 | 558 |
| Ga05G1274 | GaCBF5.4 | 47.15% | 2 | 271.5 | 97 | 180 | 20192.55 | 6.38 | Chr05 | 543 |
| Ga05G2692 | GaCBF5.5 | 53.15% | 1 | 540.0 | No Intron | 179 | 19723.45 | 5.2 | Chr05 | 540 |
| Ga06G1160 | GaCBF6.1 | 49.43% | 2 | 261.0 | 79 | 173 | 19586.52 | 9.24 | Chr06 | 522 |
| Ga07G0339 | GaCBF7.1 | 47.82% | 2 | 286.5 | 78 | 190 | 21105.74 | 6.33 | Chr07 | 573 |
| Ga07G0375 | GaCBF7.2 | 46.52% | 2 | 280.5 | 87 | 186 | 21111.88 | 9.01 | Chr07 | 561 |
| Ga07G0437 | GaCBF7.3 | 45.61% | 2 | 301.5 | 85 | 200 | 22403.13 | 6.97 | Chr07 | 603 |
| Ga07G0526 | GaCBF7.4 | 41.97% | 1 | 660.0 | No Intron | 219 | 24597.79 | 9.16 | Chr07 | 660 |
| Ga07G1656 | GaCBF7.5 | 47.06% | 1 | 714.0 | No Intron | 237 | 26067.95 | 7.67 | Chr07 | 714 |
| Ga08G2960 | GaCBF8.1 | 46.53% | 2 | 288.0 | 80 | 191 | 21147.09 | 8.94 | Chr08 | 576 |
| Ga10G0085 | GaCBF10.1 | 43.09% | 1 | 912.0 | No Intron | 303 | 32828.11 | 7.79 | Chr10 | 912 |
| Ga10G0586 | GaCBF10.2 | 49.06% | 2 | 346.5 | 938 | 230 | 25049.01 | 8.34 | Chr10 | 693 |
| Ga10G2310 | GaCBF10.3 | 46.85% | 2 | 277.5 | 338 | 184 | 20772.59 | 8.83 | Chr10 | 555 |
| Ga11G1195 | GaCBF11.1 | 43.06% | 1 | 648.0 | No Intron | 215 | 24016.73 | 5.57 | Chr11 | 648 |
| Ga11G3844 | GaCBF11.2 | 46.29% | 1 | 687.0 | No Intron | 228 | 25733.47 | 5.53 | Chr11 | 687 |
| Ga12G1792 | GaCBF12.1 | 51.35% | 1 | 555.0 | No Intron | 184 | 20415.82 | 5.6 | Chr12 | 555 |
| Ga12G1962 | GaCBF12.2 | 48.57% | 1 | 735.0 | No Intron | 244 | 27058.26 | 8.29 | Chr12 | 735 |
| Ga12G2287 | GaCBF12.3 | 50.67% | 1 | 519.0 | No Intron | 172 | 19113.28 | 9.52 | Chr12 | 519 |
| Ga13G0378 | GaCBF13.1 | 47.76% | 1 | 936.0 | No Intron | 311 | 34940.54 | 8.57 | Chr13 | 936 |
| Ga13G1841 | GaCBF13.2 | 55.49% | 1 | 501.0 | No Intron | 166 | 18268.3 | 9.16 | Chr13 | 501 |
| Ga14G0164 | GaCBF14.1 | 44.55% | 1 | 1641.0 | No Intron | 546 | 62289.41 | 8.21 | Chr14 | 1641 |
| Gthu32224 | GthCBF2.1 | 49.35% | 2 | 421.5 | 19 | 144 | 16179.23 | 5.76 | Chr02 | 843 |
| Gthu49064 | GthCBF2.2 | 47.68% | 2 | 345.0 | 361 | 268 | 30198.43 | 9.39 | Chr02 | 690 |
| Gthu13203 | GthCBF3.1 | 43.37% | 2 | 403.5 | 367 | 331 | 36123.19 | 5.6 | Chr03 | 807 |
| Gthu47888 | GthCBF4.1 | 44.54% | 2 | 348.0 | 135 | 386 | 43896.32 | 7.82 | Chr04 | 696 |
| Gthu00943 | GthCBF5.1 | 46.21% | 3 | 145.0 | 116 | 217 | 24054.12 | 7.73 | Chr05 | 435 |
| Gthu22835 | GthCBF5.2 | 45.58% | 2 | 249.0 | 163 | 265 | 29265.87 | 9.17 | Chr05 | 498 |
| Gthu23487 | GthCBF5.3 | 44.19% | 2 | 525.0 | 34 | 168 | 18527.08 | 5.07 | Chr05 | 1050 |
| Gthu27224 | GthCBF5.4 | 49.72% | 3 | 177.0 | 167.5 | 223 | 25670.21 | 6 | Chr05 | 531 |
| Gthu39378 | GthCBF6.1 | 43.36% | 2 | 429.0 | 79 | 176 | 19718.68 | 9.63 | Chr06 | 858 |
| Gthu25743 | GthCBF7.1 | 48.25% | 2 | 399.0 | 78 | 190 | 21058.73 | 7.69 | Chr07 | 798 |
| Gthu26008 | GthCBF7.2 | 49.75% | 3 | 199.0 | 242 | 231 | 26284.18 | 9.41 | Chr07 | 597 |
| Gthu26114 | GthCBF7.3 | 47.25% | 2 | 327.0 | 3590 | 402 | 45591.53 | 9.09 | Chr07 | 654 |
| Gthu26310 | GthCBF7.4 | 49.11% | 2 | 450.0 | 557 | 165 | 18298.44 | 6.16 | Chr07 | 900 |
| Gthu26345 | GthCBF7.5 | 49.30% | 2 | 357.0 | 84 | 200 | 22299.96 | 6.23 | Chr07 | 714 |
| Gthu36970 | GthCBF7.6 | 44.64% | 5 | 134.4 | 858.5 | 349 | 38963.01 | 9.23 | Chr07 | 672 |
| Gthu37544 | GthCBF10.1 | 43.55% | 1 | 744.0 | No Intron | 237 | 25840.57 | 5.11 | Chr10 | 744 |
| Gthu38206 | GthCBF10.2 | 48.69% | 2 | 381.0 | 55 | 285 | 30685.58 | 6.83 | Chr10 | 762 |
| Gthu05557 | GthCBF11.1 | 53.45% | 2 | 253.5 | 387 | 247 | 27759.34 | 6.22 | Chr11 | 507 |
| Gthu30944 | GthCBF11.3 | 44.62% | 2 | 580.5 | 26 | 229 | 25535.48 | 6.46 | Chr11 | 1161 |
| Gthu08285 | GthCBF12.1 | 45.61% | 3 | 201.0 | 105 | 198 | 22261.97 | 5.75 | Chr12 | 603 |
| Gthu09310 | GthCBF12.2 | 47.99% | 2 | 286.5 | 83 | 280 | 30538.94 | 5.41 | Chr12 | 573 |
| Gthu09767 | GthCBF12.3 | 53.65% | 2 | 192.0 | 331 | 127 | 13771.7 | 10.83 | Chr12 | 384 |
| Gthu15143 | GthCBF12.4 | 47.29% | 3 | 332.0 | 217 | 253 | 27751.02 | 5.47 | Chr12 | 996 |
| Gthu17439 | GthCBF12.5 | 50.00% | 1 | 623.0 | No Intron | 217 | 49789.68 | 5.2 | Chr12 | 623 |
| Gthu45148 | GthCBF13.1 | 45.57% | 2 | 604.5 | 48 | 299 | 33212.42 | 7.01 | Chr13 | 1209 |
| D5.v1.pred_00000002-RA | GraCBF5.1 | 48.44% | 2 | 112.5 | 25 | 74 | 8295.45 | 9.51 | Chr05 | 225 |
| D5.v1.pred_00000410-RA | GraCBF5.2 | 52.21% | 1 | 429.0 | No Intron | 142 | 15704.37 | 7.77 | Chr05 | 429 |
| D5.v1.pred_00004434-RA | GraCBF5.3 | 52.82% | 2 | 274.5 | 990 | 182 | 20389.66 | 8.73 | Chr05 | 549 |
| D5.v1.pred_00005959-RA | GraCBF11.2 | 42.84% | 1 | 663.0 | No Intron | 220 | 24580.45 | 5.83 | Chr11 | 663 |
| D5.v1.pred_00007973-RA | GraCBF11.1 | 51.20% | 1 | 498.0 | No Intron | 165 | 18295.36 | 4.83 | Chr11 | 498 |
| D5.v1.pred_00011998-RA | GraCBF8.1 | 46.88% | 2 | 288.0 | 83 | 191 | 21118.05 | 8.71 | Chr08 | 576 |
| D5.v1.pred_00012073-RA | GraCBF10.2 | 43.23% | 1 | 909.0 | No Intron | 302 | 32763.04 | 6.83 | Chr10 | 909 |
| D5.v1.pred_00012733-RA | GraCBF10.3 | 49.58% | 1 | 714.0 | No Intron | 237 | 25768.47 | 5.11 | Chr10 | 714 |
| D5.v1.pred_00014544-RA | GraCBF10.1 | 46.77% | 2 | 279.0 | 331 | 185 | 20936.8 | 8.85 | Chr10 | 558 |
| D5.v1.pred_00017398-RA | GraCBF12.2 | 46.84% | 1 | 822.0 | No Intron | 273 | 30483.42 | 9.21 | Chr12 | 822 |
| D5.v1.pred_00018348-RA | GraCBF12.1 | 48.69% | 1 | 957.0 | No Intron | 318 | 35178.55 | 9.26 | Chr12 | 957 |
| D5.v1.pred_00019084-RA | GraCBF13.1 | 48.67% | 1 | 1056.0 | No Intron | 351 | 39106.31 | 9.4 | Chr13 | 1056 |
| D5.v1.pred_00024969-RA | GraCBF3.2 | 47.57% | 2 | 309.0 | 33 | 205 | 22488.63 | 4.49 | Chr03 | 618 |
| D5.v1.pred_00026415-RA | GraCBF3.1 | 50.28% | 1 | 537.0 | No Intron | 178 | 19754.22 | 5.33 | Chr03 | 537 |
| D5.v1.pred_00033697-RA | GraCBF7.4 | 47.82% | 2 | 286.5 | 78 | 190 | 20945.69 | 6.84 | Chr07 | 573 |
| D5.v1.pred_00033730-RA | GraCBF7.1 | 46.23% | 2 | 318.0 | 87 | 211 | 23734.29 | 10.38 | Chr07 | 636 |
| D5.v1.pred_00033795-RA | GraCBF7.3 | 45.44% | 2 | 301.5 | 84 | 200 | 22316.95 | 6.23 | Chr07 | 603 |
| D5.v1.pred_00033875-RA | GraCBF7.2 | 44.33% | 1 | 609.0 | No Intron | 202 | 22778.41 | 8.5 | Chr07 | 609 |
| D5.v1.pred_00033889-RA | GraCBF7.5 | 43.14% | 1 | 663.0 | No Intron | 220 | 24862.16 | 9.3 | Chr07 | 663 |
| D5.v1.pred_00036818-RA | GraCBF4.1 | 45.40% | 2 | 472.5 | 87 | 314 | 35900.04 | 6.52 | Chr04 | 945 |
| D5.v1.pred_00039026-RA | GraCBF2.1 | 43.34% | 1 | 999.0 | No Intron | 332 | 37235.74 | 6.27 | Chr02 | 999 |
| TURN.00g264260 | TurnCBF10.2 | 43.20% | 1 | 912.0 | No Intron | 303 | 32831.1 | 6.3 | Chr10 | 912 |
| TURN.00g270000 | TurnCBF10.3 | 49.78% | 1 | 675.0 | No Intron | 224 | 24304.86 | 5.09 | Chr10 | 675 |
| TURN.00g286990 | TurnCBF10.1 | 47.13% | 2 | 279.0 | 329 | 185 | 20951.81 | 8.62 | Chr10 | 558 |
| TURN.01g298290 | TurnCBF11.1 | 47.02% | 1 | 687.0 | No Intron | 228 | 25671.53 | 5.79 | Chr11 | 687 |
| TURN.01g324440 | TurnCBF11.2 | 43.10% | 1 | 645.0 | No Intron | 214 | 23884.55 | 5.83 | Chr11 | 645 |
| TURN.02g031330 | TurnCBF2.2 | 48.72% | 1 | 507.0 | No Intron | 168 | 18382.47 | 9.24 | Chr02 | 507 |
| TURN.02g032640 | TurnCBF2.1 | 45.47% | 1 | 783.0 | No Intron | 260 | 29083.66 | 6.31 | Chr02 | 783 |
| TURN.02g338020 | TurnCBF12.2 | 48.65% | 1 | 816.0 | No Intron | 271 | 29624.05 | 6.14 | Chr12 | 816 |
| TURN.02g342270 | TurnCBF12.5 | 50.29% | 1 | 513.0 | No Intron | 170 | 18789.92 | 9.52 | Chr12 | 513 |
| TURN.02g345760 | TurnCBF12.3 | 48.44% | 1 | 735.0 | No Intron | 244 | 27170.5 | 8.75 | Chr12 | 735 |
| TURN.02g347630 | TurnCBF12.4 | 51.53% | 1 | 555.0 | No Intron | 184 | 20383.77 | 5.6 | Chr12 | 555 |
| TURN.02g360310 | TurnCBF12.1 | 46.62% | 1 | 828.0 | No Intron | 275 | 31361.13 | 9.83 | Chr12 | 828 |
| TURN.03g053990 | TurnCBF3.1 | 47.54% | 1 | 873.0 | No Intron | 290 | 31434.64 | 4.91 | Chr03 | 873 |
| TURN.03g067920 | TurnCBF3.2 | 50.84% | 1 | 537.0 | No Intron | 178 | 19740.19 | 5.33 | Chr03 | 537 |
| TURN.03g370380 | TurnCBF13.1 | 50.00% | 1 | 864.0 | No Intron | 287 | 31758.52 | 5.99 | Chr13 | 864 |
| TURN.03g396620 | TurnCBF13.2 | 46.88% | 2 | 288.0 | 82 | 191 | 21148.07 | 8.71 | Chr13 | 576 |
| TURN.04g098300 | TurnCBF4.1 | 43.80% | 1 | 669.0 | No Intron | 222 | 25121.76 | 4.98 | Chr04 | 669 |
| TURN.05g105390 | TurnCBF5.5 | 48.51% | 1 | 672.0 | No Intron | 205 | 23049.81 | 7.73 | Chr05 | 672 |
| TURN.05g121830 | TurnCBF5.1 | 53.15% | 1 | 540.0 | No Intron | 179 | 19753.47 | 5.07 | Chr05 | 540 |
| TURN.05g121840 | TurnCBF5.6 | 54.34% | 1 | 357.0 | No Intron | 119 | 12834.49 | 9.72 | Chr05 | 357 |
| TURN.05g136300 | TurnCBF5.3 | 46.41% | 2 | 271.5 | 97 | 180 | 20244.67 | 6.38 | Chr05 | 543 |
| TURN.05g143120 | TurnCBF5.4 | 46.59% | 2 | 279.0 | 101 | 185 | 21126.98 | 8.44 | Chr05 | 558 |
| TURN.05g147530 | TurnCBF5.2 | 47.93% | 2 | 301.5 | 81 | 200 | 22130.7 | 7.78 | Chr05 | 603 |
| TURN.06g164340 | TurnCBF6.1 | 49.53% | 2 | 265.5 | 79 | 176 | 19745.67 | 9.54 | Chr06 | 531 |
| TURN.07g187610 | TurnCBF7.3 | 47.06% | 1 | 714.0 | No Intron | 237 | 26142.96 | 6.97 | Chr07 | 714 |
| TURN.07g199780 | TurnCBF7.6 | 42.84% | 1 | 663.0 | No Intron | 220 | 24818.15 | 9.43 | Chr07 | 663 |
| TURN.07g199920 | TurnCBF7.2 | 45.20% | 1 | 531.0 | No Intron | 176 | 19892.94 | 5.7 | Chr07 | 531 |
| TURN.07g200710 | TurnCBF7.4 | 45.44% | 2 | 301.5 | 84 | 200 | 22299.96 | 6.23 | Chr07 | 603 |
| TURN.07g201330 | TurnCBF7.1 | 46.17% | 2 | 280.5 | 88 | 186 | 21081.9 | 9.17 | Chr07 | 561 |
| TURN.07g201650 | TurnCBF7.5 | 48.34% | 2 | 286.5 | 78 | 190 | 21003.65 | 6.84 | Chr07 | 573 |
| Golon.002G040200-1 | GolonCBF2.1 | 40.90% | 1 | 1005.0 | No Intron | 334 | 35263.92 | 6.6 | Chr02 | 1005 |
| Golon.002G072400-1 | GolonCBF2.2 | 35.00% | 1 | 1283.0 | No Intron | 226 | 24798.56 | 4.94 | Chr02 | 1283 |
| Golon.003G252100-1 | GolonCBF3.1 | 41.80% | 1 | 1799.0 | No Intron | 302 | 32864.55 | 7.65 | Chr03 | 1799 |
| Golon.004G175100-1 | GolonCBF4.1 | 45.20% | 1 | 969.0 | No Intron | 322 | 36814.11 | 5.67 | Chr04 | 969 |
| Golon.005G017000-1 | GolonCBF5.1 | 47.40% | 3 | 218.0 | 116 | 217 | 24240.31 | 7.02 | Chr05 | 654 |
| Golon.005G061200-1 | GolonCBF5.3 | 46.06% | 2 | 279.0 | 101 | 185 | 21150.11 | 8.79 | Chr05 | 558 |
| Golon.005G127900-1 | GolonCBF5.2 | 45.99% | 2 | 324.0 | 97 | 215 | 24229.29 | 7.88 | Chr05 | 648 |
| Golon.005G263600-1 | GolonCBF5.4 | 43.36% | 1 | 918.0 | No Intron | 179 | 19723.45 | 5.2 | Chr05 | 918 |
| Golon.006G017300-1 | GolonCBF6.2 | 47.59% | 1 | 519.0 | No Intron | 172 | 19249.8 | 9.9 | Chr06 | 519 |
| Golon.006G046700-1 | GolonCBF6.1 | 46.45% | 1 | 648.0 | No Intron | 215 | 23862.96 | 6.9 | Chr06 | 648 |
| Golon.007G035000-1 | GolonCBF7.5 | 47.99% | 2 | 286.5 | 78 | 190 | 21106.72 | 5.97 | Chr07 | 573 |
| Golon.007G038300-1 | GolonCBF7.1 | 46.70% | 2 | 280.5 | 88 | 186 | 21081.86 | 9.01 | Chr07 | 561 |
| Golon.007G043700-1 | GolonCBF7.4 | 45.11% | 2 | 301.5 | 77 | 200 | 22395.15 | 6.92 | Chr07 | 603 |
| Golon.007G051500-1 | GolonCBF7.2 | 44.18% | 1 | 636.0 | No Intron | 211 | 24155.68 | 5.68 | Chr07 | 636 |
| Golon.007G053000-1 | GolonCBF7.6 | 41.55% | 1 | 657.0 | No Intron | 218 | 24554.76 | 9.32 | Chr07 | 657 |
| Golon.007G175300-1 | GolonCBF7.3 | 40.95% | 1 | 1216.0 | No Intron | 183 | 20406.72 | 5.2 | Chr07 | 1216 |
| Golon.008G292900-1 | GolonCBF8.1 | 40.11% | 2 | 647.0 | 82 | 191 | 21148.07 | 8.71 | Chr08 | 1294 |
| Golon.010G082000-1 | GolonCBF10.1 | 47.21% | 2 | 277.5 | 306 | 184 | 20854.78 | 8.81 | Chr10 | 555 |
| Golon.010G247700-1 | GolonCBF10.3 | 44.01% | 1 | 1018.0 | No Intron | 234 | 25435.15 | 5.23 | Chr10 | 1018 |
| Golon.010G299700-1 | GolonCBF10.2 | 38.71% | 1 | 1483.0 | No Intron | 348 | 38285.38 | 8.53 | Chr10 | 1483 |
| Golon.011G286100-1 | GolonCBF11.1 | 40.06% | 1 | 1348.0 | No Intron | 215 | 24028.73 | 6.2 | Chr11 | 1348 |
| Golon.012G025600-1 | GolonCBF12.1 | 48.86% | 1 | 837.0 | No Intron | 278 | 30323.86 | 5.75 | Chr12 | 837 |
| Golon.012G122200-1 | GolonCBF12.2 | 42.63% | 1 | 1004.0 | No Intron | 272 | 30366.17 | 8.94 | Chr12 | 1004 |
| Golon.012G141800-1 | GolonCBF12.3 | 45.03% | 1 | 886.0 | No Intron | 185 | 20668.34 | 5.83 | Chr12 | 886 |
| Golon.013G042500-1 | GolonCBF13.1 | 49.01% | 1 | 861.0 | No Intron | 286 | 31787.63 | 5.6 | Chr13 | 861 |
| Golon.013G190200-1 | GolonCBF13.2 | 39.23% | 1 | 1147.0 | No Intron | 166 | 18293.36 | 7.77 | Chr13 | 1147 |
| EPI10_000250 | GauCBF1.1 | 40.61% | 1 | 879.0 | No Intron | 292 | 32615.61 | 5.26 | Chr01 | 879 |
| EPI10_004288 | GauCBF2.1 | 46.58% | 1 | 453.0 | No Intron | 150 | 16707.79 | 5.51 | Chr02 | 453 |
| EPI10_004656 | GauCBF3.1 | 48.78% | 1 | 861.0 | No Intron | 286 | 30682.01 | 5.37 | Chr03 | 861 |
| EPI10_007995 | GauCBF4.1 | 45.40% | 1 | 1077.0 | No Intron | 358 | 40867.96 | 6.81 | Chr04 | 1077 |
| EPI10_009003 | GauCBF5.1 | 51.05% | 1 | 621.0 | No Intron | 206 | 23053.82 | 7.74 | Chr05 | 621 |
| EPI10_012710 | GauCBF7.1 | 45.99% | 1 | 561.0 | No Intron | 186 | 21069.84 | 8.99 | Chr07 | 561 |
| EPI10_012803 | GauCBF7.2 | 44.68% | 1 | 555.0 | No Intron | 184 | 20988.83 | 4.74 | Chr07 | 555 |
| EPI10_012759 | GauCBF7.3 | 44.94% | 1 | 603.0 | No Intron | 200 | 22364.18 | 7.77 | Chr07 | 603 |
| EPI10_012684 | GauCBF7.4 | 46.85% | 1 | 555.0 | No Intron | 184 | 20643.29 | 8.44 | Chr07 | 555 |
| EPI10_012816RB | GauCBF7.5 | 42.63% | 1 | 624.0 | No Intron | 207 | 23454.59 | 9.75 | Chr07 | 624 |
| EPI10_014119 | GauCBF8.1 | 46.70% | 1 | 576.0 | No Intron | 191 | 21148.07 | 8.71 | Chr08 | 576 |
| EPI10_019934 | GauCBF10.1 | 46.92% | 1 | 552.0 | No Intron | 183 | 20675.37 | 8.61 | Chr10 | 552 |
| EPI10_034045 | GauCBF13.1 | 48.26% | 1 | 603.0 | No Intron | 200 | 22150.68 | 6.54 | Chr13 | 603 |
| EPI10_033448 | GauCBF13.2 | 46.24% | 1 | 558.0 | No Intron | 185 | 21085.93 | 8.79 | Chr13 | 558 |
| EPI10_033558 | GauCBF13.3 | 51.52% | 1 | 429.0 | No Intron | 142 | 15676.36 | 7.76 | Chr13 | 429 |
| Chr: chromosome; GC: Guanine-cytosine | | | | | | | | | | |
