# Supplementary material for: Functional Characterization of Cotton C-Repeat Binding Factor Genes Reveal Their Potential Role in Cold Stress Tolerance
Source: Front Plant Sci. 2021 Dec 8;12:766130. doi: 10.3389/fpls.2021.766130 (PMC8692369; doi:10.3389/fpls.2021.766130)
Supplement: Supplementary file 2 [file Table_2.DOCX]

Table S2. Subcellular localization of the proteins encoded by the CBL genes

| Gene name | Gene Annotation | Nucleus | Cytolsol | Plasma membrane | Mitochondrion | Chloroplast | Cyto_nucl | Cyto_mito | Chlo_mito | Endoplasmic reticulum | Golgi apparatus | Vaculolar membrane | Extracellular | E.R._plas | pero |
| --- | --- | --- | --- | --- | --- | --- | --- | --- | --- | --- | --- | --- | --- | --- | --- |
| Ghe02G18690 | GheCBF2.1 | 11 | 1 | 1 | 0 | 1 | 0 | 0 | 0 | 0 | 0 | 0 | 0 | 0 | 0 |
| Ghe02G19970 | GheCBF2.2 | 11 | 3 | 0 | 0 | 0 | 0 | 0 | 0 | 0 | 0 | 0 | 0 | 0 | 0 |
| Ghe03G03390 | GheCBF3.1 | 0 | 0 | 0 | 5 | 9 | 0 | 0 | 0 | 0 | 0 | 0 | 0 | 0 | 0 |
| Ghe03G07060 | GheCBF3.2 | 12.5 | 0 | 1 | 0 | 0 | 7 | 0 | 0 | 0 | 0 | 0 | 0 | 0 | 0 |
| Ghe04G19140 | GheCBF4.1 | 13 | 0 | 1 | 0 | 0 | 0 | 0 | 0 | 0 | 0 | 0 | 0 | 0 | 0 |
| Ghe05G12970 | GheCBF5.5 | 10.5 | 0 | 1.5 | 0 | 0 | 6 | 0 | 0 | 0 | 0 | 0 | 0 | 0 | 0 |
| Ghe05G06300 | GheCBF5.4 | 1 | 13 | 0 | 0 | 0 | 0 | 0 | 0 | 0 | 0 | 0 | 0 | 0 | 0 |
| Ghe05G27290 | GheCBF5.2 | 3 | 0 | 0 | 2.5 | 8 | 0 | 2 | 0 | 0 | 0 | 0 | 0 | 0 | 0 |
| Ghe05G03790 | GheCBF5.1 | 2.5 | 0 | 0 | 7 | 4 | 2 | 0 | 0 | 0 | 0 | 0 | 0 | 0 | 0 |
| Ghe05G01760 | GheCBF5.3 | 8 | 0 | 2 | 4 | 0 | 0 | 0 | 0 | 0 | 0 | 0 | 0 | 0 | 0 |
| Ghe06G13000 | GheCBF6.1 | 3 | 0 | 0 | 10.5 | 0 | 0 | 0 | 6 | 0 | 0 | 0 | 0 | 0 | 0 |
| Ghe07G18320 | GheCBF7.4 | 2 | 8 | 0 | 1 | 3 | 0 | 0 | 0 | 0 | 0 | 0 | 0 | 0 | 0 |
| Ghe07G03800 | GheCBF7.5 | 11 | 0 | 0 | 0 | 1 | 0 | 0 | 0 | 0 | 0 | 0 | 0 | 0 | 0 |
| Ghe07G04380 | GheCBF7.2 | 3 | 0 | 0 | 9 | 2 | 0 | 0 | 0 | 0 | 0 | 0 | 0 | 0 | 0 |
| Ghe07G01750 | GheCBF7.3 | 11 | 0 | 1.5 | 1 | 0 | 0 | 0 | 0 | 0 | 0 | 0 | 0 | 0 | 0 |
| Ghe07G03420 | GheCBF7.6 | 8 | 6 | 0 | 0 | 0 | 0 | 0 | 0 | 0 | 0 | 0 | 0 | 0 | 0 |
| Ghe07G05270 | GheCBF7.1 | 4 | 0 | 0 | 0 | 8.5 | 0 | 1.3 | 5.3 | 0 | 0 | 0 | 0 | 0 | 0 |
| Ghe08G32310 | GheCBF8.1 | 6 | 0 | 0 | 1 | 6 | 0 | 0 | 0 | 0 | 0 | 0 | 0 | 0 | 0 |
| Ghe09G06510 | GheCBF9.1 | 12 | 1 | 0 | 1 | 0 | 0 | 0 | 0 | 0 | 0 | 0 | 0 | 0 | 0 |
| Ghe10G08080 | GheCBF10.1 | 9 | 0 | 0 | 2 | 3 | 0 | 0 | 0 | 0 | 0 | 0 | 0 | 0 | 0 |
| Ghe10G32620 | GheCBF10.3 | 9 | 0 | 0 | 3 | 2 | 0 | 0 | 0 | 0 | 0 | 0 | 0 | 0 | 0 |
| Ghe10G27020 | GheCBF10.2 | 12.5 | 0 | 1 | 0 | 0 | 7 | 0 | 0 | 0 | 0 | 0 | 0 | 0 | 0 |
| Ghe11G03020 | GheCBF11.1 | 13 | 1 | 0 | 0 | 0 | 0 | 0 | 0 | 0 | 0 | 0 | 0 | 0 | 0 |
| Ghe11G31060 | GheCBF11.2 | 7 | 0 | 0 | 3 | 4 | 0 | 0 | 0 | 0 | 0 | 0 | 0 | 0 | 0 |
| Ghe12G07790 | GheCBF12.1 | 12 | 2 | 0 | 0 | 0 | 0 | 0 | 0 | 0 | 0 | 0 | 0 | 0 | 0 |
| Ghe12G15310 | GheCBF12.3 | 13 | 1 | 0 | 0 | 0 | 0 | 0 | 0 | 0 | 0 | 0 | 0 | 0 | 0 |
| Ghe12G12080 | GheCBF12.2 | 12 | 1 | 0 | 1 | 0 | 0 | 0 | 0 | 0 | 0 | 0 | 0 | 0 | 0 |
| Ghe13G04590 | GheCBF13.1 | 5 | 0 | 0 | 3 | 6 | 0 | 0 | 0 | 0 | 0 | 0 | 0 | 0 | 0 |
| Ghe13G19950 | GheCBF13.2 | 9 | 0 | 0 | 0 | 5 | 0 | 0 | 0 | 0 | 0 | 0 | 0 | 0 | 0 |
| Gthu00943 | GthCBF5.1 | 0 | 2 | 0 | 0 | 0 | 0 | 0 | 0 | 5.5 | 1.5 | 2 | 3 | 4 | 0 |
| Gthu05557 | GthCBF11.1 | 6 | 2 | 0 | 5 | 0 | 0 | 0 | 0 | 0 | 0 | 0 | 1 | 0 | 0 |
| Gthu08285 | GthCBF12.1 | 0 | 1 | 4 | 0 | 0 | 0 | 0 | 0 | 0 | 0 | 7 | 2 | 0 | 0 |
| Gthu09310 | GthCBF12.2 | 6 | 2 | 0 | 5 | 0 | 0 | 0 | 0 | 0 | 0 | 0 | 1 | 0 | 0 |
| Gthu09767 | GthCBF12.3 | 6 | 2 | 0 | 5 | 0 | 0 | 0 | 0 | 0 | 0 | 0 | 1 | 0 | 0 |
| Gthu13203 | GthCBF3.1 | 0 | 1 | 4 | 0 | 0 | 0 | 0 | 0 | 0 | 0 | 7 | 2 | 0 | 0 |
| Gthu15143 | GthCBF12.4 | 6 | 2 | 0 | 5 | 0 | 0 | 0 | 0 | 0 | 0 | 0 | 1 | 0 | 0 |
| Gthu22835 | GthCBF5.2 | 0 | 1 | 7 | 0 | 0 | 0 | 0 | 0 | 0 | 0 | 5 | 1 | 0 | 0 |
| Gthu23487 | GthCBF5.3 | 6 | 2 | 0 | 5 | 0 | 0 | 0 | 0 | 0 | 0 | 0 | 1 | 0 | 0 |
| Gthu25743 | GthCBF7.1 | 6 | 2 | 0 | 5 | 0 | 0 | 0 | 0 | 0 | 0 | 0 | 1 | 0 | 0 |
| Gthu26008 | GthCBF7.2 | 6 | 2 | 0 | 5 | 0 | 0 | 0 | 0 | 0 | 0 | 0 | 1 | 0 | 0 |
| Gthu26114 | GthCBF7.3 | 0 | 1 | 7 | 0 | 0 | 0 | 0 | 0 | 0 | 0 | 5 | 1 | 0 | 0 |
| Gthu26310 | GthCBF7.4 | 0 | 2 | 0 | 2 | 0 | 0 | 0 | 0 | 4.5 | 1.5 | 0 | 4 | 3.5 | 0 |
| Gthu26345 | GthCBF7.5 | 0 | 2 | 0 | 2 | 0 | 0 | 0 | 0 | 5.5 | 1.5 | 0 | 3 | 4 | 0 |
| Gthu27224 | GthCBF5.4 | 6 | 2 | 0 | 5 | 0 | 0 | 0 | 0 | 0 | 0 | 0 | 1 | 0 | 0 |
| Gthu30944 | GthCBF11.3 | 6 | 2 | 0 | 5 | 0 | 0 | 0 | 0 | 0 | 0 | 0 | 1 | 0 | 0 |
| Gthu32224 | GthCBF2.1 | 5 | 2 | 0 | 6 | 0 | 0 | 0 | 0 | 0 | 0 | 0 | 1 | 0 | 0 |
| Gthu36970 | GthCBF7.6 | 6 | 2 | 0 | 5 | 0 | 0 | 0 | 0 | 0 | 0 | 0 | 1 | 0 | 0 |
| Gthu37544 | GthCBF10.1 | 6 | 2 | 0 | 5 | 0 | 0 | 0 | 0 | 0 | 0 | 0 | 1 | 0 | 0 |
| Gthu38206 | GthCBF10.2 | 0 | 1 | 5 | 0 | 0 | 0 | 0 | 0 | 0 | 0 | 4 | 4 | 0 | 0 |
| Gthu39378 | GthCBF6.1 | 6 | 1 | 0 | 5 | 0 | 0 | 0 | 0 | 0 | 0 | 0 | 2 | 0 | 0 |
| Gthu45148 | GthCBF13.1 | 0 | 1 | 7 | 0 | 0 | 0 | 0 | 0 | 0 | 0 | 4 | 0 | 0 | 0 |
| Gthu47888 | GthCBF4.1 | 0 | 1 | 11 | 0 | 0 | 0 | 0 | 0 | 0 | 1 | 1 | 0 | 0 | 0 |
| Gthu49064 | GthCBF2.2 | 0 | 4.5 | 1.5 | 2 | 0 | 0 | 0 | 0 | 3.5 | 0 | 0 | 1 | 3 | 0 |
| Gthu17439 | GthCBF12.5 | 14 | 0 | 0 | 0 | 0 | 0 | 0 | 0 | 0 | 0 | 0 | 0 | 0 | 0 |
| EPI10_009003 | GauCBF5.1 | 14 | 0 | 0 | 0 | 0 | 0 | 0 | 0 | 0 | 0 | 0 | 0 | 0 | 0 |
| EPI10_004288 | GauCBF2.1 | 12.5 | 0 | 0 | 1 | 0 | 0 | 7 | 0 | 0 | 0 | 0 | 0 | 0 | 0 |
| EPI10_012684 | GauCBF7.4 | 8 | 0 | 0 | 4 | 0 | 1 | 0 | 0 | 0 | 0 | 0 | 0 | 0 | 0 |
| EPI10_012710 | GauCBF7.1 | 3 | 0 | 0 | 9 | 2 | 0 | 0 | 0 | 0 | 0 | 0 | 0 | 0 | 0 |
| EPI10_012759 | GauCBF7.3 | 10 | 0 | 1.5 | 1 | 1 | 0 | 0 | 0 | 0 | 0 | 0 | 0 | 0 | 0 |
| EPI10_012803 | GauCBF7.2 | 12.5 | 0 | 0 | 1 | 0 | 0 | 7 | 0 | 0 | 0 | 0 | 0 | 0 | 0 |
| EPI10_012816RB | GauCBF7.5 | 2 | 2 | 0 | 0 | 0 | 0 | 0 | 0 | 0 | 0 | 0 | 0 | 0 | 10 |
| EPI10_007995 | GauCBF4.1 | 10 | 1 | 1 | 0 | 2 | 0 | 0 | 0 | 0 | 0 | 0 | 0 | 0 | 0 |
| EPI10_014119 | GauCBF8.1 | 6 | 0 | 0 | 1 | 6 | 1 | 0 | 0 | 0 | 0 | 0 | 0 | 0 | 0 |
| EPI10_019934 | GauCBF10.1 | 8 | 0 | 0 | 1 | 5 | 0 | 0 | 0 | 0 | 0 | 0 | 0 | 0 | 0 |
| EPI10_004656 | GauCBF3.1 | 5 | 1 | 0 | 1 | 7 | 0 | 0 | 0 | 0 | 0 | 0 | 0 | 0 | 0 |
| EPI10_033448 | GauCBF13.2 | 2 | 0 | 0 | 9.5 | 2.5 | 0 | 0 | 6.5 | 0 | 0 | 0 | 0 | 0 | 0 |
| EPI10_033558 | GauCBF13.3 | 1 | 13 | 0 | 0 | 0 | 0 | 0 | 0 | 0 | 0 | 0 | 0 | 0 | 0 |
| EPI10_034045 | GauCBF13.1 | 10.5 | 0 | 1.5 | 0 | 0 | 1 | 6 | 0 | 0 | 0 | 0 | 0 | 0 | 0 |
| EPI10_000250 | GauCBF1.1 | 12.5 | 1.5 | 0 | 0 | 0 | 0 | 7.5 | 0 | 0 | 0 | 0 | 0 | 0 | 0 |
