# Supplementary material for: Functional Characterization of Cotton C-Repeat Binding Factor Genes Reveal Their Potential Role in Cold Stress Tolerance
Source: Front Plant Sci. 2021 Dec 8;12:766130. doi: 10.3389/fpls.2021.766130 (PMC8692369; doi:10.3389/fpls.2021.766130)
Supplement: Supplementary file 3 [file Table_3.DOCX]

Table S3: List of primers for RT-qPCR analysis

| No. | Seq ID | Orientation | Len | Seq |
| --- | --- | --- | --- | --- |
| 1 | Gthu25743 | FORWARD | 20 | AACGGGCAAAATGCGAAGAC |
| 2 | Gthu25743 | REVERSE | 21 | GCATTATCGGTGTCCAGCCT |
| 3 | Gthu45148 | FORWARD | 20 | GTTTTGGGATGGAATGGGCG |
| 4 | Gthu45148 | REVERSE | 23 | GCTTCCAGTTCTTTACACTAGCA |
| 5 | Gthu08285 | FORWARD | 20 | CCGAATCCGCCATGAAACAC |
| 6 | Gthu08285 | REVERSE | 20 | AAACAAAACGCCGCTACGTC |
| 7 | Gthu22835 | FORWARD | 20 | GTGAGGGTTCGGCTGTATCT |
| 8 | Gthu22835 | REVERSE | 20 | AAACCCCATTGCAGCTAGGA |
| 9 | Gthu15143 | FORWARD | 21 | GGCGCAATTAGAGTGTCATGC |
| 10 | Gthu15143 | REVERSE | 20 | GGACCCGAAACGGAGAATGT |
| 11 | Gthu30944 | FORWARD | 21 | ACCTTCCGGATCTGTTGATGC |
| 12 | Gthu30944 | REVERSE | 21 | CTCCCTAAGGCGCGACTAATA |
| 13 | Gthu36970 | FORWARD | 22 | TGCAGAGTATTGCATTTGCTCG |
| 14 | Gthu36970 | REVERSE | 21 | TTCGAAGGCTCAGTTCCTGTT |
| 15 | Gthu32224 | FORWARD | 21 | GGCGTATTCGACGGTATCTCA |
| 16 | Gthu32224 | REVERSE | 21 | TCCCAAAGGCGAGGCTCATTA |
| 17 | Gthu09767 | FORWARD | 19 | GCGGCTCTCTCGGGAAAAA |
| 18 | Gthu09767 | REVERSE | 21 | CCTCCACTACAGTGTGCATGA |
| 19 | Gthu26114 | FORWARD | 20 | CGTCGGGGGTATGTTTAACG |
| 20 | Gthu26114 | REVERSE | 20 | GTGGCAAAGTCACCGAGAAG |
| 21 | Gthu23487 | FORWARD | 19 | GAGCCTTGGTCCGGCTATC |
| 22 | Gthu23487 | REVERSE | 20 | CAACCCCACACATCATTGGC |
| 23 | Gthu27224 | FORWARD | 20 | ACAATCAGTGCAGGAGCCAA |
| 24 | Gthu27224 | REVERSE | 20 | GCGAACTCCCGTCTCACTAC |
| 25 | Ghe12G12080 | FORWARD | 20 | TGGGTGGCTGAGATTAGGGA |
| 26 | Ghe12G12080 | REVERSE | 20 | ACGGTCCACGCAGGTAAAAA |
| 27 | Ghe07G03420 | FORWARD | 20 | AACGGGCAAAATGCGAAGAC |
| 28 | Ghe07G03420 | REVERSE | 20 | GTCGGTGTCCAGCCTTAGAC |
| 29 | Ghe05G06300 | FORWARD | 20 | TAGAGGTAGTGGGACGGGAG |
| 30 | Ghe05G06300 | REVERSE | 20 | TGAACGGCTGGGTTCTTTCT |
| 31 | Ghe12G15310 | FORWARD | 20 | CCACTGCTCGTGACATCCAA |
| 32 | Ghe12G15310 | REVERSE | 20 | GGCGAAAGTCCATCCACAAG |
| 33 | Ghe13G04590 | FORWARD | 20 | CGTTTCCCTGGTGACTTCCA |
| 34 | Ghe13G04590 | REVERSE | 20 | TACTAAACATCGTCGTCGCC |
| 35 | Ghe07G18320 | FORWARD | 20 | CAAAAACACTTCCACGCCCC |
| 36 | Ghe07G18320 | REVERSE | 20 | ACTTGTTCTTGCCTCGGTCG |
| 37 | Ghe05G27290 | FORWARD | 20 | AAGAGGCAGCATTGAGGCTT |
| 38 | Ghe05G27290 | REVERSE | 20 | CATCCACATCTTGGGCGAGT |
| 39 | Ghe12G07790 | FORWARD | 20 | CTCTTCACCCATGACGACGA |
| 40 | Ghe12G07790 | REVERSE | 20 | ATCCCAAACGAACAGCTCCT |
| 41 | Ghe09G06510 | FORWARD | 21 | TCCGGTTTCTACTGATCCCAA |
| 42 | Ghe09G06510 | REVERSE | 21 | CATTAGCTTCAGAGTTCCCCT |
| 43 | Ghe05G12970 | FORWARD | 20 | TAGACCACAGCAGCGATACC |
| 44 | Ghe05G12970 | REVERSE | 20 | TTGCCCCACACATAAGCCTC |
| 45 | Ghe04G19140 | FORWARD | 20 | TTTGGGGTGGAAGTGGAACT |
| 46 | Ghe04G19140 | REVERSE | 20 | TTGATGGTGAGGGCTACGGA |
| 47 | Ghe03G03390 | FORWARD | 20 | CACGCTGGCATTCCTACAAA |
| 48 | Ghe03G03390 | REVERSE | 20 | GCAGGCTCTGACTTTAGGCT |
| 49 | Ghe02G18690 | FORWARD | 19 | CAGGTTGCTTCCATGGCTA |
| 50 | Ghe02G18690 | REVERSE | 21 | CACACCCCTATAAAGCTTCGC |
| 51 | Ghe07G03800 | FORWARD | 20 | TCAGTCGTCGTCATCCAAGC |
| 52 | Ghe07G03800 | REVERSE | 20 | GCGCCTTATTTTGTGGCTCC |
| 53 | Ghe10G08080 | FORWARD | 21 | CGCAAATGTTGCAAAACTCCA |
| 54 | Ghe10G08080 | REVERSE | 20 | TCCCTGCTCTCTTTTGCCAC |
| 55 | EPI10_033448 | FORWARD | 20 | GCAGGAGCCAAAAAGTGAGC |
| 56 | EPI10_033448 | REVERSE | 20 | AAGAGACTTGAACGGCTGGG |
| 57 | EPI10_012684 | FORWARD | 20 | AGAAGGGTATGGTTGGGCAC |
| 58 | EPI10_012684 | REVERSE | 20 | CTTCGCATTTTGCCCGTTCA |
| 59 | EPI10_004288 | FORWARD | 20 | CCGGTAAGTTCATCGCCCAA |
| 60 | EPI10_004288 | REVERSE | 20 | GAGATACCGTCGAAGACGCC |
| 61 | EPI10_012803 | FORWARD | 20 | ATGCGAAGTTGGGGCAAATG |
| 62 | EPI10_012803 | REVERSE | 21 | AGCAGGACGAGGAAGTTGATG |
| 63 | EPI10_033558 | FORWARD | 20 | CCGGATATAGCAGACGCCAA |
| 64 | EPI10_033558 | REVERSE | 20 | ACAGACCCACCGCCAAAATA |
| 65 | EPI10_004656 | FORWARD | 20 | CCCCGACAAATACCACGACA |
| 66 | EPI10_004656 | REVERSE | 20 | AAGCTCAGTCCCTAACCCCA |
| 67 | EPI10_000250 | FORWARD | 23 | GGGAAATGGGTTAGTGAAGTGAG |
| 68 | EPI10_000250 | REVERSE | 21 | ATCTCAGCAGCTGGACATGAA |
| 69 | EPI10_007995 | FORWARD | 19 | AGCGAGCCAGTGTTTTGTC |
| 70 | EPI10_007995 | REVERSE | 20 | GTTGACGTTGAGGGCTACGG |
| 71 | EPI10_012816RB | FORWARD | 22 | CAACTGAATCCCAACCCTCGAA |
| 72 | EPI10_012816RB | REVERSE | 20 | GCTGAAATGTGGGAGTGAGT |
| 73 | EPI10_019934 | FORWARD | 20 | CTCAGATATCCGCCACCCTT |
| 74 | EPI10_019934 | REVERSE | 20 | TGGGGACTGGGAAGTTTGTT |
| 75 | EPI10_014119 | FORWARD | 20 | AGTGGGGCTCTTGGGTATCT |
| 76 | EPI10_014119 | REVERSE | 20 | TTGTGAGCAGTAGCAGGAGG |
| 77 | EPI10_012710 | FORWARD | 20 | ATGACGGCATTGCAGGGAAG |
| 78 | EPI10_012710 | REVERSE | 20 | AGGTTTGAACTGCTGGGTGG |
| 79 | EPI10_012759 | FORWARD | 20 | GGAGACCCAAAAGGCACTGT |
| 80 | EPI10_012759 | REVERSE | 20 | GGTGCCTTGCTGCATTTTCT |
| 81 | RD29A | FORWARD | 22 | CTTGTCGACGAGAAGCAAAGAA |
| 82 | RD29A | REVERSE | 22 | TCTTGATGGAGAATTCGTGTCC |
| 83 | COR15A | FORWARD | 22 | GTCGTCGTTTCTCAACGCAAGA |
| 84 | COR15A | REVERSE | 22 | GCTTTCTCAGCTTCTTTACCCA |
| 85 | KIN1 | FORWARD | 22 | ATGCCTTCCAAGCCGGTCAGAC |
| 86 | KIN1 | REVERSE | 22 | CCGGTCTTGTCCTTCACGAAGT |
| 87 | COR47 | FORWARD | 22 | TGTCATCGAAAAGCTTCACCGA |
| 88 | COR47 | REVERSE | 22 | ACCGGGATGGTAGTGGAAACTG |
| 89 | GthuCBF4 | FORWARD | 20 | CGTGGCAGCTATAGCACTGA |
| 90 | GthuCBF4 | REVERSE | 20 | TTCTGAAAGTCTCCGCCACC |
| 91 | GhActin | FORWARD | 19 | ATCCTCCGTCTTGACCTTG |
| 92 | GhActin | REVERSE | 19 | TGTCCGTCAGGCAACTCAT |
